# Genomic associations with bill length and disease reveal drift and selection across island bird populations

**DOI:** 10.1002/evl3.38

**Published:** 2018-01-26

**Authors:** Claire Armstrong, David S. Richardson, Helen Hipperson, Gavin J. Horsburgh, Clemens Küpper, Lawrence Percival‐Alwyn, Matt Clark, Terry Burke, Lewis G. Spurgin

**Affiliations:** ^1^ School of Biological Sciences, University of East Anglia Norwich Research Park Norwich NR4 7TJ United Kingdom; ^2^ NERC Biomolecular Analysis Facility, Department of Animal and Plant Sciences University of Sheffield Sheffield S10 2TN United Kingdom; ^3^ Max Planck Institute for Ornithology 82319 Seewiesen Germany; ^4^ Earlham Institute Norwich Research Park Norwich NR4 7UZ United Kingdom

**Keywords:** Adaptation, birds, GWAS, population genomics

## Abstract

Island species provide excellent models for investigating how selection and drift operate in wild populations, and for determining how these processes act to influence local adaptation and speciation. Here, we examine the role of selection and drift in shaping genomic and phenotypic variation across recently separated populations of Berthelot's pipit (*Anthus berthelotii*), a passerine bird endemic to three archipelagos in the Atlantic. We first characterized genetic diversity and population structuring that supported previous inferences of a history of recent colonizations and bottlenecks. We then tested for regions of the genome associated with the ecologically important traits of bill length and malaria infection, both of which vary substantially across populations in this species. We identified a SNP associated with variation in bill length among individuals, islands, and archipelagos; patterns of variation at this SNP suggest that both phenotypic and genotypic variation in bill length is largely shaped by founder effects. Malaria was associated with SNPs near/within genes involved in the immune response, but this relationship was not consistent among archipelagos, supporting the view that disease resistance is complex and rapidly evolving. Although we found little evidence for divergent selection at candidate loci for bill length and malaria resistance, genome scan analyses pointed to several genes related to immunity and metabolism as having important roles in divergence and adaptation. Our findings highlight the utility and challenges involved with combining association mapping and population genetic analysis in nonequilibrium populations, to disentangle the effects of drift and selection on shaping genotypes and phenotypes.

**Impact Summary**

Since the time of Darwin, evolutionary biologists have sought to understand how natural selection and random chance act to shape the extraordinary diversity we see in nature. Natural selection acts on individual differences that have a genetic basis, and these differences accumulate until new species eventually form. To fully grasp how natural selection operates in the wild, we need to understand the link between variation at the level of genes, individuals, populations, and species. In this study, we used genetic analyses to test how differences have evolved among island populations of Berthelot's pipit—a small passerine bird endemic to three North Atlantic archipelagos. Berthelot's pipits are separated into subspecies based on differences in bill length, and island populations differ markedly in their levels of disease. Until now, however, we did not know whether these differences were the product of natural selection, or random processes that are the feature of evolutionary histories of small island populations. We found a genetic variant associated with bill length in Berthelot's pipits, which is close to a gene involved in controlling bill shape in Darwin's finches. Importantly, we showed that variation in this genomic region—and therefore variation in bill length—is a product of the colonization history of the pipits. In addition, we identified two genes that may be associated with malaria infection—an ecologically important disease in animals. However, this association varied among islands, suggesting that resistance to malaria is not fixed, but constantly changing, as expected for host–pathogen coevolution. Finally, our analyses suggest that genes related to immune function and metabolism may under natural selection, which is consistent with studies on humans and other animals. Our study shows that combining genetic techniques with ecological study is a powerful way to understand how natural selection acts in island populations.

The process of speciation is a continuum, beginning with small genetic differences between lineages that accumulate to produce reproductive isolation (Coyne and Orr 2004). Studying the traits that diverge in the early stages of speciation will increase our understanding of the factors and forces that give rise to the huge array of species we see today. Phenotypic divergence between populations may be driven by natural selection acting upon adaptive traits, by genetic drift mediated by demographic forces, or by a combination of the two. To determine whether variation in traits across populations is adaptive, it is first necessary to understand the demographic history of the diverging populations to separate the relative contribution of drift. Molecular markers have been used since the 1960s to characterize genetic diversity within and among populations (Lewontin and Hubby 1966; Kreitman 1983), identify demographic patterns (Jorde et al. 1997; Garris et al. 2005), and infer adaptation (Hughes and Nei 1988; Zhang et al. 1998), and therefore have been instrumental in the study of reproductive isolation and speciation.

Within the last decade, next‐generation sequencing has greatly enhanced our ability to study evolutionary patterns and processes in wild populations (Stapley et al. 2010). Using techniques such as restriction site‐associated DNA sequencing (RAD‐seq; Miller et al. 2007; Baird et al. 2008), it is possible to sequence thousands of loci without any prior knowledge of the target species’ genome (Davey et al. 2011). The high marker density provides enough power to identify subtle population differentiation and calculate accurate genome‐wide estimates of variation (Luikart et al. 2003; Corander et al. 2013; Benestan et al. 2015; Fernández et al. 2016). It has also facilitated the identification of individual loci or regions of the genome directly under natural selection. “Top‐down” approaches, such as genome‐wide association studies and quantitative trait locus mapping, enable the discovery of regions of the genome associated with ecologically important traits (e.g., Jones et al. 2012; Küpper et al. 2015; Liu et al. 2015; Pavey et al. 2015; Comeault et al. 2016). Alternatively, using “bottom‐up” (or genome‐scanning) approaches, it is possible to statistically detect loci under divergent selection between populations, and to use this information to identify candidate phenotypes previously not known to be under selection (Turner et al. 2010; Fumagalli et al. 2015; Kardos et al. 2015; Manel et al. 2016). Combining trait mapping and genome scans is a particularly powerful approach, as it potentially allows us to simultaneously identify novel candidate traits under selection, determine the genetic basis of traits, and investigate how those traits have evolved over ecological and evolutionary timescales (Stinchcombe and Hoekstra 2008; Gagnaire and Gaggiotti 2016; Josephs et al. 2017). A number of studies have now combined top‐down and bottom‐up approaches to study adaptation in the wild (Nadeau et al. 2012; Gompert et al. 2013; Johnston et al. 2014; Brachi et al. 2015; Bourgeois et al. 2017), but few have been carried out in bottlenecked or isolated populations (Hohenlohe et al. 2010).

Oceanic island systems provide excellent models in which to study adaptive evolution. Populations on different islands will face a unique set of selection pressures, driven by variation in abiotic factors, or differences in ecological communities between islands that have arisen due to independent histories of colonization and extinction events (Carlquist 1974). The sea provides a natural barrier to the migration of terrestrial fauna and flora, enabling populations to independently evolve, with limited scope for gene flow to counteract local adaptation (Grant 1986).

Here, we combine association mapping and genome‐scanning approaches to identify the key traits involved in adaptive evolution and population divergence in Berthelot's pipit (*Anthus berthelotii*), a small passerine endemic to three volcanic island archipelagos in the Atlantic Ocean. The recent colonization of this species across a set of ecologically distinct islands makes it an interesting model for examining adaptive evolution in a natural setting. Within the last 2.5 million years Berthelot's pipits colonized the Canary Islands and diverged from their sister species, the tawny pipit (*Anthus campestris*; Voelker 1999). Then, around 8000 years ago, Berthelot's pipits from the Canary Islands reached the Madeiran and Selvagens islands in two independent, northward colonizations, with a subsequent absence of gene flow (Illera et al. 2007; Spurgin et al. 2014). The recent colonization of the latter two archipelagos has resulted in clear genetic and phenotypic signatures of founder effects (Spurgin et al. 2014; González‐Quevedo et al. 2015b). This nested pattern of population structure enables comparisons of genetic and phenotypic divergence at a range of spatial scales (Spurgin et al. 2014; González‐Quevedo et al. 2015a), and allows us to examine how natural selection and genetic drift act in bottlenecked populations (Spurgin et al. 2011).

Importantly, in Berthelot's pipits there is also inter‐island variation in selection pressures and potential signatures of adaptation (reviewed in Illera et al. 2016). Two traits of particular interest are disease resistance and bill morphology. The different pipit populations have a high degree of variation in avian malaria prevalence, and this variation is consistent over time and constrained by island biogeographic features, suggesting that avian malaria may be an important agent of divergent selection in this system (Illera et al. 2008; Spurgin et al. 2012; González‐Quevedo et al. 2014). Bill morphology also differs markedly among populations, with pipits from the Madeiran archipelago classified as a separate subspecies, *A. berthelotii madeirensis*, based on longer bill lengths (Martín and Lorenzo 2001; Oliveira and Menezes 2004). Although overall patterns of body size in Berthelot's pipits are likely to have been shaped by founder effects (Spurgin et al. 2014), the difference in bill length could be adaptive. Importantly, both disease resistance and bill morphology are key traits in local adaptation, divergence, and speciation in other taxa (Amadon 1950; Grant 1986; Ekblom et al. 2007; Eizaguirre et al. 2012; Lenz et al. 2013; Lamichhaney et al. 2016; Bosse et al. 2017), and as a result there is a great deal of interest in identifying their genetic basis in wild populations. Indeed, characterizing the molecular basis of these traits increases our understanding of the mechanisms driving adaptive evolution, and could contribute to improving biodiversity conservation efforts (Smith et al. 2009).

Our specific aims were as follows. First, we used RAD‐seq data to reexamine the population history of Berthelot's pipit at greater resolution than previously achieved with microsatellites (Spurgin et al. 2014). Second, we use association mapping to identify candidate SNPs associated with bill length and malaria infection among the “founder” Berthelot's pipit populations in the Canary Islands. We then explore how selection and drift have interacted to shape variation at candidate SNPs by comparing variation among founder and derived bottlenecked populations. Finally, we performed genome scans to detect regions of the genome under divergent selection between populations and examine how drift and selection shape genome diversity in bottlenecked populations.

## Methods

### SAMPLE COLLECTION AND SEQUENCING

**Figure 1:**
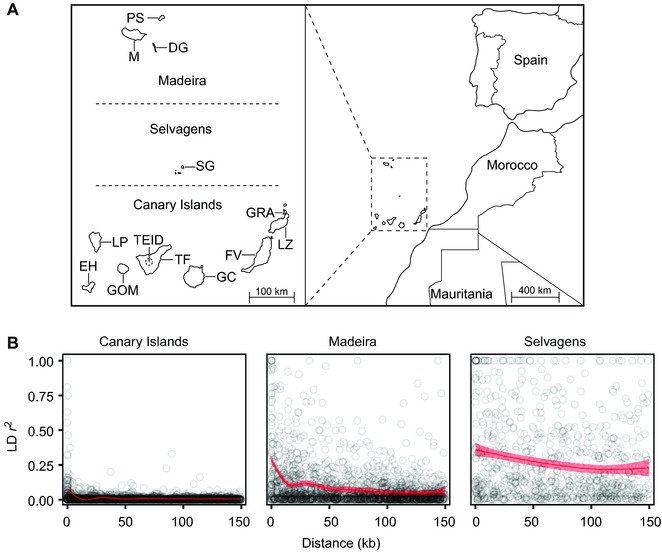
Sampling locations, genetic diversity, and linkage disequilibrium in Berthelot's and tawny pipits. (A) Map of Berthelot's pipit and tawny pipit sampling locations. (B) The relationship between linkage disequilibrium and base‐pair distance for SNPs in the Canary Islands, Madeira, and Selvagens archipelagos. The red line shows a general additive model, with the shaded band indicating 95% confidence intervals.

Berthelot's pipits were sampled on 12 islands across their geographical range (Fig. 1A), as reported by Illera et al. (2007) and Spurgin et al. (2012). Pipits caught on the alpine plateau of El Teide volcano (>2000 m above sea level) were classified as a separate population (Spurgin et al. 2014). Full sampling details are provided in the Supplementary Methods.

We chose 20 samples per population (22 from the lowland Tenerife population) for RAD‐seq, with efforts made to reduce the probability of sampling relatives by maximizing geographical coverage within each island, and to equalize the number of males and females where possible. In addition, 16 samples from tawny pipits were included; these were collected in Spain (*n* = 11), Mauritania (*n* = 4), and Morocco (*n* = 1; Fig. 1A) following the same catching, measuring, and sampling protocols as Berthelot's pipit. Sequencing and bioinformatics were performed using the ddRAD protocol by DaCosta and Sorenson (2014), with loci mapped to the zebra finch genome (*Taeniopygia guttata*, version 3.2.4; Warren et al. 2010). Where initial mapping was unsuccessful, a draft Berthelot's pipit genome was used to improve mapping to the zebra finch genome (see Supplementary Methods). Following demultiplexing (Supplementary Methods), samples were grouped into five datasets, as outlined in Table 1. These were created to maximize the number of loci at different levels of clustering within our dataset (Supplementary Methods; Table 1).

**Table 1:** Summary of RAD‐Seq datasets: Sample locations, locus filtering, and analyses performed on each dataset

	Dataset					
	All pipits	Berthelot's	Berthelot's HWE	Canary Islands	Madeira	Selvagens
*n* Samples	278	262	262	182	60	20
*n* Loci	1826	2745	2598	3330	2983	1918
Read depth	184	198	198	187	195	176
Density of loci in zebra finch genome (%)	0.016	0.025	0.024	0.030	0.027	0.017
*Sample locations*						
Canary Islands	X	X	X	X		
Madeira	X	X	X		X	
Selvagens	X	X	X			X
Tawny pipits	X					
*Filtering steps*						
Filtering A	X					
Filtering B		X	X	X	X	X
Filtering C		X	X	X	X	X
Filtering D			X			
*Analyses*						
Nucleotide diversity	X					
Linkage disequilibrium				X	X	X
Admixture			X			
BSLMM/LMM		X		X		
EigenGWAS		X				

Read depth = median number of reads per sample per RAD locus. Filtering steps: A = loci unambiguously genotyped in 100% of samples, excluding loci with multiallelic SNPs; B = loci with ≤3 ambiguous genotypes, and up to 10% missing/ambiguous genotypes; C = remove SNPs with minor allele frequency of <3%, then select one SNP per locus with the highest minor allele frequency; D = remove SNPs out of Hardy–Weinberg equilibrium in more than two populations, and SNPs mapped to the Z chromosome.

### POPULATION GENETICS ANALYSIS

We first compared the overall levels of genetic diversity among the different Berthelot's pipit archipelagos and the tawny pipit, using the “All Pipits” dataset. We used the approach outlined by Lozier (2014) to calculate per‐SNP nucleotide diversity (π_SNP_) at 1826 loci present in all samples, and the overall nucleotide diversity per locus (π_RAD_) as the sum of the nucleotide diversity at every site along a RAD locus, including invariable sites, divided by the length of the locus (excluding loci < 50 bp long, *n* = 1722). Nucleotide diversity was calculated in VCFtools version 0.1.14015 (Danecek et al. 2011). To account for differences in sample size between groups, nucleotide diversity for each SNP was averaged across 100 runs using subsampled datasets of 16 samples, equal to the smallest sample size (tawny pipits).

Linkage disequilibrium was calculated in PLINK 1.9 (Chang et al. 2015). We used the separate archipelago datasets (“Canary Islands,” “Madeira,” and “Selvagens,”) rather than the “Berthelot's” dataset, to maximize the number of loci available for analysis. The *r* ^2^ values obtained were compared to physical distance between loci, excluding pairs of SNPs situated on different chromosomes.

Population structure was examined using the “Berthelot's HWE” dataset. Genetic population clustering was identified with ADMIXTURE (Alexander et al. 2009), for *K* = 1 to *K* = 13 putative clusters. We calculated fivefold cross‐validation error to determine the optimal number of clusters (Alexander and Lange 2011). PLINK 1.9 (Chang et al. 2015) was used to calculate the mean *F*_ST_ between each pair of populations, which we correlated with pairwise geographic distance using a one‐tailed partial Mantel test, using the Ecodist package in R (Goslee and Urban 2007). To control for archipelago‐level effects, we included “archipelago comparison” (e.g., Canary Islands vs Canary Islands, Canary Islands vs Madeira, etc.) as a categorical variable in the partial Mantel tests.

### GENETIC ASSOCIATIONS WITH TRAITS

We used two genome‐wide association study (GWAS) analyses to identify loci associated with bill length and malaria infection. A central issue with GWAS is accounting for population structure (Marchini et al. 2004) and we used two approaches to deal with this. First, we restricted association analyses to the “Canary Islands” dataset, in which population structure is limited (see Results). Secondly, we used kinship/relatedness matrices to statistically control for any population structure among the Canary Islands. We first used a univariate linear‐mixed model (LMM) implemented in the software package GEMMA (Zhou and Stephens 2012). This model assumes that every genetic variant affects the phenotype, which would suit a highly polygenic genetic architecture, and accounts for population structure with a centered kinship matrix calculated in GEMMA.

In our second analysis, we performed association mapping with a Bayesian sparse linear‐mixed model (BSLMM; Zhou et al. 2013), again using GEMMA and the “Canary Islands” dataset. BSLMM combines linear‐mixed models with sparse regression models, giving the benefits of each when the underlying genetic architecture of the trait (many genes of small effect vs few genes of large effect) is unknown (Zhou et al. 2013). Population structure is controlled for with a relatedness matrix as a covariate of the model. The output includes estimates of the hyperparameters PVE (the proportion of the phenotypic variance explained), PGE (the proportion of PVE that can be explained by SNPs with a nonzero effect on phenotypic variation), and the number of SNPs that explain the PVE. Each SNP is assigned a posterior inclusion probability (PIP), which is the proportion of times the SNP has a nonzero effect on phenotypic variation. We used a threshold of PIP > 0.1 to identify candidate SNPs associated with phenotypes (Chaves et al. 2016). Each analysis was run for 20 million iterations with a burn‐in of 5 million. This was repeated 10 times, and the results were averaged across runs. We subsequently repeated the BSLMM analysis using the “Berthelot's” dataset, to discover whether the increase in power gained from a larger sample size improved the ability to detect loci associated with either bill length or malaria infection.

To check whether SNPs associated with bill length also covary with overall body size, we also ran a BSLMM with tarsus length as a candidate trait. Tarsus length is the strongest predictor of overall body size that can be accurately measured from a live bird (Senar and Pascual 1997). Bill and tarsus length were only weakly correlated in our dataset (*r* = 0.13, *P* = 0.04).

To identify genes located near outlier SNPs, we downloaded Ensembl gene predictions for zebra finch (http://ftp://ftp.ensembl.org/pub/release-90/fasta/taeniopygia_guttata/pep/), and viewed regions of interest using the University of Santa Cruz Genome Browser (http://genome-euro.ucsc.edu/cgi-bin/hgGateway).

We examined how selection and drift shaped variation at candidate SNPs, as follows. For the most significant SNPs identified in the BSLMM analyses (PIP > 0.1), we calculated allele frequencies for each island population and compared this against population‐level trait variation (mean bill length and malaria prevalence), using Pearson correlations. We also calculated pairwise *F*_ST_ individually for BSLMM outliers, and compared outlier *F*_ST_ and genome‐wide *F*_ST_, using partial Mantel tests. To better visualize how variation at outlier SNPs was partitioned among populations, we also tested for isolation‐by distance at outlier SNPs, using the same approach described above for genome‐wide *F*_ST_.

### EigenGWAS ANALYSIS

We used EigenGWAS (Chen et al. 2016), implemented in the program GEAR (https://github.com/gc5k/GEAR/wiki), to separate loci under divergent selection from the genome‐wide effects of drift between the archipelagos, using the “Berthelot's” dataset. This analysis uses eigenvector decomposition on the genotype data to characterize the underlying population genetic structuring, without the need to define discrete populations. This then enables the identification of SNPs under selection across gradients of population structure. EigenGWAS provides adjusted *P* values that use the genomic inflation factor λ_GC_ (Devlin and Roeder 1999) to correct for population stratification (i.e., drift), to avoid interpreting ancestry‐informative markers as loci under selection. Genes in the zebra finch genome located near to outlier SNPs were identified as above. We then calculated *F*_ST_ in PLINK 1.9 (Chang et al. 2015), grouping samples according to eigenvector clustering, to examine the relationship between EigenGWAS significance and genetic divergence. Finally, we tested whether significant BSLMM and EigenGWAS SNPs were more closely located than expected by chance using randomization tests (Supplementary Methods).

## Results

### RAD LIBRARIES AND GENOME SUMMARY

The three RAD libraries produced 277,627,890 (first), 202,466,438 (second), and 184,660,891 (third) raw reads with GC contents of 52%, 51% and 50%, respectively. Following filtering, we produced six datasets containing between 1826 and 3330 polymorphic RAD loci per dataset, at a median read depth of between 176 and 198 reads per sample per locus (Table 1).

We created a draft Berthelot's pipit genome that we used to improve our ability to map RAD loci to the zebra finch genome. We used DISCOVAR de novo (Weisenfeld et al. 2014) to produce an assembly 1,153,192,274 bp long (94.3% of the length of the zebra finch genome), with a contig N50 of 355,835 bp (see Supplementary Results and Table S1 for further details).

### GENETIC DIVERSITY AND POPULATION STRUCTURE

We used two measures of nucleotide diversity, π_SNP_ and π_RAD_, to compare genetic diversity between the three separate Berthelot's pipit archipelagos and tawny pipits (Table S2, Fig. S1). In line with expectations based on population sizes, tawny pipits had the highest levels of diversity (π_SNP_ = 0.104, π_RAD_ = 0.0049), followed by Berthelot's pipit populations in the Canary Islands (π_SNP_ = 0.012, π_RAD_ = 0.0005), Madeira (π_SNP_ = 0.008, π_RAD_ = 0.0004), and Selvagens (π_SNP_ = 0.006, π_RAD_ = 0.0003). There was moderate divergence between Deserta Grande and the rest of the Madeiran archipelago (see below), however, exclusion of Deserta Grande had little impact on measures of diversity (Table S2).

This trend was reflected throughout Berthelot's pipit archipelagos for linkage disequilibrium (LD; Fig. 1B). Baseline levels of LD were lowest in the Canary Islands and highest in Selvagens, with the rate of decay sharpest in the Canary Islands and shallowest in Selvagens.

**Figure 2:**
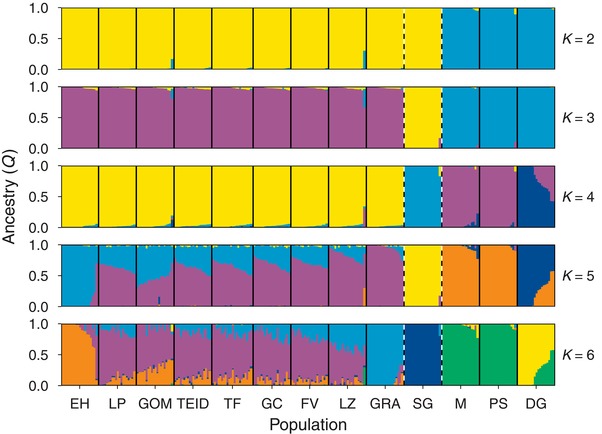
Admixture analysis at *K* = 2 to *K* = 6 clusters for Berthelot’s pipit. Vertical bars represent individual pipits, and are colored by their assignment to each one of *K* clusters. Solid black lines indicate separate populations; dashed black lines indicate separate archipelagos. Populations from left to right: Canary Islands – El Hierro (EH), La Palma (LP), Gomera (GOM), Teide (TEID), Tenerife (TF), Gran Canaria (GC), Fuerteventura (FV), Lanzarote (LZ), Graciosa (GRA); Selvagens – Selvagem Grande (SG); Madeira – Madeira (M), Porto Santo (PS), Deserta Grande (DG).

Population admixture analysis (Fig. 2) first separated Madeira from the Canary Islands and Selvagens (*K* = 2) and then into the three archipelagos (*K* = 3). Further population structuring beyond the archipelago level was seen with Deserta Grande diverging from the other islands in the Madeiran archipelago (*K* = 4), and an east–west gradient in admixture across the Canary Islands (*K* = 5; Fig. 2). The most likely number of clusters determined by cross‐validation error was four (Fig. S2), however similar cross‐validation errors were found for *K* = 3 (0.434), *K* = 4 (0.431), and *K* = 5 (0.434). ADMIXTURE plots for *K* = 7 to *K* = 13 are shown in Fig. S3. We found a moderate signal of isolation by distance within the Canary Island archipelago (Mantel test, *r* = 0.37, *P* = 0.002; Fig. S4), although levels of structure within the archipelago were generally low (*F*_ST_ < 0.03).

### ASSOCIATION STUDIES

Both LMMs and BSLMMs were used to find regions of the genome associated with malaria and bill length in the “Canary Islands” dataset, while accounting for population structure. The LMM analysis failed to find any SNPs significantly associated with either malaria or bill length when using a Bonferroni‐corrected significance threshold of *P* < 1.5 × 10^−5^; however, Bonferroni correction is often overly conservative, inflating the risk of type II errors (Johnson et al. 2010). There was strong concordance between LMM *P* values and BSLMM PIPs for malaria (Pearson's correlation between –log LMM *P* values and log BSLMM PIPs, *r* = 0.91, *P* < 0.0001) and bill length (*r* = 0.91, *P* < 0.0001).

**Figure 3:**
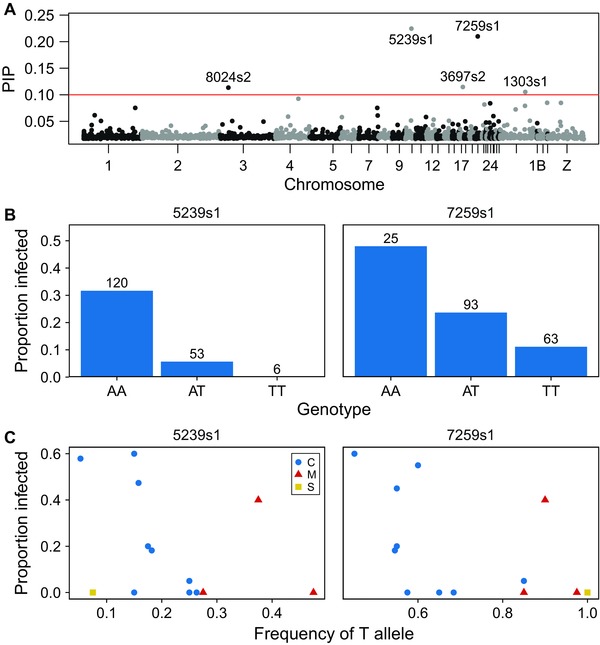
Genetic associations with malaria infection. (A) Manhattan plot of BSLMM analysis of malaria infection in the Canary Islands. Red line indicates the threshold of posterior inclusion probability (PIP) = 0.1. For the top two outlier SNPs: (B) the association between genotype and the proportion of individuals infected with malaria in the Canary Islands. Numbers above bars indicate the number of individuals with each genotype; and (C) per‐population minor allele frequency and the proportion of infected individuals, colored by archipelago (C = Canary Islands, M = Madeira, S = Selvagens).

In the BSLMM analysis for malaria, a median of 68.4% of phenotypic variation was explained by the genotype (95% CI 11.2–99.8%), of which 22.5% was explained by SNPs with nonzero effects, but the credible intervals on this estimate were very high (95% CI 0.0–91.3%). Five SNPs were found with a PIP > 0.1, after controlling for population structure (Fig. 3A). Of these outlier SNPs, the top two also had the smallest *P* values in the LMM (Table 2). The strongest association was found for a SNP on chromosome 10 (5239s1), approximately 2000 bp from the *IL‐16* gene. The next strongest association was with a SNP on chromosome 20 (7259s1) situated within an intronic region of *RIMS4*. Both of these SNPs had “A” and “T” alleles. The “AA” and “TT” homozygotes were associated with the highest and lowest levels of malaria, respectively, while heterozygotes had intermediate malaria prevalence (Fig. 3B). We then examined how population‐level allele frequencies at these SNPs were related to population‐level prevalence of malaria. Within the Canary Islands, there was a clear correlation between malaria prevalence and allele frequency for SNP 5239s1 (*r* = –0.76, *P* = 0.02); however, this pattern was not observed when the populations from the bottlenecked northern archipelagos were included (*r* = –0.35, *P* = 0.25; Fig. 3C). Despite showing an individual‐level association between malaria prevalence and genotype (Fig. 3B), the population‐level allele frequency at SNP 7259s1 was not significantly correlated with malaria infection in either the Canary Islands (*r* = –0.59; *P* = 0.09) or across all populations (*r* = –0.50; *P* = 0.09; Fig. 3C). Estimates of *F*_ST_ revealed that patterns of structure at both of these SNPs was correlated with genome‐wide *F*_ST_ across all Berthelot's pipit populations (partial Mantel tests, 5239s1: *r* = 0.40, *P* = 0.007; 7259s1: *r* = 0.46; *P* = 0.01; Fig. S5A). Outlier *F*_ST_ values were correlated with pairwise geographic distance (5239s1: *r* = 0.30, *P* = 0.009; 7259s1: *r* = 0.36; *P* = 0.01; Fig. S5B).

**Table 2:** Outlier SNPs with PIP > 0.1 in BSLMM analyses for bill length and malaria

Phenotype	SNP	Locus ID	PIP	LMM *P*	Position	Gene	Distance (bp)
Bill length	8914s1	4494	0.10	0.0005	Chr5:60950937	–	–
Malaria	5239s1	2739	0.22	0.0001	Chr10:12048280	*IL‐16*	2188
	7259s1	3870	0.21	0.0002	Chr20:6483195	*RIMS4*	In gene
	3697s2	4893	0.11	0.002	Chr17:8691633	–	–
	8024s2	4199	0.11	0.002	Chr3:18288583	*CNIH4*	1964
	1303s1	632	0.11	0.0008	Chr1A:50095389	*CACNA1I*	In gene

The *P* value from the LMM analyses is shown. The nearest gene within 10,000 bp of the SNP is identified.

**Figure 4:**
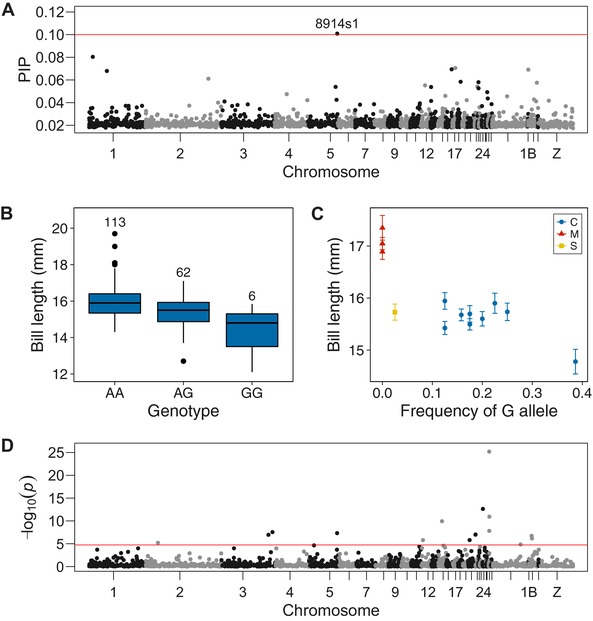
Genetic associations with bill length and selection across archipelagos. (A) Manhattan plot of BSLMM analysis of bill length in the Canary Islands. Red line indicates the threshold of posterior inclusion probability (PIP) = 0.1. For the top outlier SNP: (B) the association between genotype and bill length in the Canary Islands. Numbers above box plots indicate the number of individuals with each genotype; and (C) per‐population minor allele frequency and mean bill length, colored by archipelago (C = Canary Islands, M = Madeira, S = Selvagens). (D) Manhattan plot of EigenGWAS eigenvector 1. Red line indicates the Bonferroni‐corrected *P* value threshold.

The BSLMM for bill length found that genotype explained a median of 67.9% of phenotypic variation (95% CI 14.0–99.7%), with 14.3% explained by SNPs with nonzero effects (although again the latter had wide credible intervals; 0.0–85.6%). The strongest association with bill length was for a SNP on chromosome 5 (Fig. 4A; Table 2). At this SNP (8914s1), individual bill length decreases with increasing number of copies of the “G” allele, and heterozygotes had intermediate bill length (Fig. 4B). This region was not associated with tarsus length (Fig. S6A), and there were no SNPs that showed strong associations for both bill length and tarsus length (Fig. S6B). At the population level, mean bill length decreases with increasing frequency of the “G” allele at the most significant BSLMM SNP, both in the Canary Islands (*r* = –0.69, *P* = 0.04), and across all Berthelot's pipit populations (*r* = –0.82, *P* = 0.0006; Fig. 4C). After controlling for archipelago‐level effects, patterns of *F*_ST_ at this SNP were strongly correlated with genome‐wide *F*_ST_ (*r* = 0.64, *P* = 0.002; Fig. S5A), and with geographic distance (*r* = 0.48, *P* = 0.002; Fig. S5B).

Repeating the BSLMM analyses using the “Berthelot's” dataset resulted in higher PIPs for the outlier SNPs mentioned above, but also identified a number of additional outlier loci (Supplementary Results, Table S3, Fig. S7).

### DIFFERENTIAL SELECTION ACROSS ARCHIPELAGOS

We used EigenGWAS to detect loci putatively under divergent selection between the archipelagos in the “Berthelot's” dataset. The first eigenvector (EV1) separated the Madeiran archipelago from the Canary Islands and Selvagens, and the second (EV2) separated Selvagens from Madeira and the Canary Islands (Fig. S8A). The first two eigenvectors captured a large proportion of the genetic variation, with eigenvalues of 31.1 and 14.6, respectively. Both EV1 and EV2 had considerable genomic inflation factors (26.5 and 9.7, respectively), suggesting high levels of population structuring (a genomic inflation factor ≤ 1 indicates no population structuring; Hinrichs et al. 2009), so we used the adjusted *P* values calculated by EigenGWAS that use genomic control to account for population structure.

Using a Bonferroni‐corrected *P* < 1.8 × 10^−5^, we detected signatures of selection in EV1 at 21 SNPs, 16 of which could be mapped to the zebra finch genome (Fig. 4D; Table 3). A larger number of outliers was found along EV2, with 44 outlier SNPs (33 mapped) between Selvagens and the other archipelagos (Fig. S8B; Table S4). A comparison of EigenGWAS *P* values and *F*_ST_ between eigenvector clusters shows that highly significant EigenGWAS SNPs always had high *F*_ST_, whereas a substantial number of SNPs with high *F*_ST_ were nonsignificant in the EigenGWAS analysis (Fig. S8C).

**Table 3:** Outlier SNPs from Eigenvector 1 of EigenGWAS analysis

SNP	Locus ID	*P*	Position	Gene	Distance (bp)
203s1	105	6 × 10^−26^	Chr27:1229392	*MAP3K14*	In gene
7826s2	4142	2 × 10^−13^	Chr24:1583716	*DLAT*	In gene
7622s1	4065	1 × 10^−11^	Chr27:1534267	*GPATCH8*	965
5483s1	2876	4 × 10^−11^	–	–	–
8418s1	4348	1 × 10^−10^	Chr14:7479033	*SNX29*	In gene
2982s2	1469	2 × 10^−10^	–	–	–
2041s1	997	1 × 10^−9^	–	–	–
174s1	85	2 × 10^−8^	Chr27:1376142	*MYO1D*	In gene
5752s1	3039	3 × 10^−8^	Chr3:105320550	–	–
8322s2	4296	5 × 10^−8^	Chr5:58018667	*AP5M1*	2868
7216s3	3844	7 × 10^−8^	–	–	–
4131s1	2115	9 × 10^−8^	Chr20:15447166	*PREX1*	In gene
2473s1	1217	1 × 10^−7^	Chr20:15293405	*CSE1L*	In gene
6932s2	3689	1 × 10^−7^	Chr3:96985953	–	–
7180s1	3825	2 × 10^−7^	Chr4A:5912527	–	–
1728s2	849	6 × 10^−7^	–	–	–
6765s1	3602	7 × 10^−7^	Chr4A:6655419	*VSIG4*	In gene
4138s2	2120	2 × 10^−6^	Chr20:3241763	*GSS*	4
1723s1	846	2 × 10^−6^	Chr12:3914968	–	–
1541s1	760	6 × 10^−6^	Chr2:25304006	*VPS50*	In gene
2273s1	1117	1 × 10^−5^	Chr1A:56816580	–	–

The nearest gene within 10,000 bp of the SNP is identified.

None of the candidate SNPs identified in the BSLMM analyses was identified as outliers in the EigenGWAS analysis. An outlier SNP in the EigenGWAS analysis of EV1 was located close to the region on chromosome 5 that showed an association with bill length (Fig. 4). However, closer inspection of the region revealed that the most significant SNP was 2,932,270 bp from the most significant BSLMM SNP (Fig. S9), which was not closer than expected by chance (randomization test, *P* = 0.1). A second SNP in the region also had an elevated (but not significant) PIP value in the BSLMM analysis and was much closer to the significant EigenGWAS candidate SNP (444,885 bp; Fig. S9; *P* < 0.001). The regions identified in the BSLMM for association with malaria did not show signs of divergent selection between the archipelagos.

## Discussion

Using RAD sequencing, we examined fine‐scale population structure among Berthelot's pipit populations, finding within‐archipelago genetic structuring between island populations, as well as confirming the previously detected patterns of among‐archipelago variation. After controlling for demographic history, we identified candidate SNPs associated with the ecologically important traits of bill length and malaria resistance. Examining allele frequency variation at candidate SNPs revealed that: (i) population‐level candidate SNP variation was related to population‐level trait variation for bill length both within the founder Canary Islands archipelago and throughout all archipelagos, but only within the Canary Islands for malaria; and, (ii) candidate SNP variation was correlated with genome‐wide variation. Finally, although we found little evidence for divergent selection on bill length or malaria resistance, we found signatures of divergent selection between the archipelagos across the genome, including at genes involved in metabolism and immune function.

Comparisons of genome‐wide diversity in Berthelot's and tawny pipits provided support for previous inferences of colonization history from microsatellites and mitochondrial DNA (Illera et al. 2007; Spurgin et al. 2014). The past colonization history and associated bottlenecks are reflected in linkage disequilibrium (LD) within the archipelagos, indicating a larger, more outbred population in the Canary Islands, compared with smaller, more inbred populations in Madeira and Selvagens. This is in concordance with previous estimations of effective population size using approximate Bayesian Computation, which predicted contemporary effective population sizes of ∼4000, ∼3000, and ∼400 individuals for the Canary Islands, Madeira, and Selvagens, respectively (Spurgin et al. 2014). Using population admixture analysis, we were able to further describe population structure: Deserta Grande diverged from the rest of the Madeiran archipelago, possibly suggesting a further bottleneck on Deserta Grande. We also detected a weak east–west gradient in population structure within the Canary Islands, which was supported by the finding of isolation‐by‐distance throughout this archipelago.

We next aimed to identify the genetic regions associated with bill length and malaria resistance—two traits of major evolutionary importance (Smith et al. 1995; Daszak 2000; Grant and Grant 2006). The high level of LD relative to outbred populations (e.g., Kardos et al. 2016) has enabled us to detect associations with these two polygenic traits in Berthelot's pipit, with a modest number of markers. The strongest association with bill length was found at a SNP in a region on chromosome 5. This SNP (8914s1) is not located within a gene, which is perhaps not surprising, given (i) the density of our marker set, and (ii) the fact that many SNPs associated with polygenic traits are located in regulatory regions (Maurano et al. 2012). The wider region surrounding SNP 8914s1 and a significant EigenGWAS SNP (see below) contains, among others, the genes *BMP4* and *OTX2*. *BMP4* is a clear candidate for involvement in bill morphology, as studies in Darwin's finches, chickens, and ducks have experimentally demonstrated its role in beak development (Abzhanov et al. 2004; Wu et al. 2004). *OTX2* plays a crucial role in craniofacial development across jawed vertebrates (Kimura et al. 1997), and mutations in this gene and structural variants within the wider genomic region have been linked with craniofacial abnormalities in mice and humans (Hide et al. 2002; Zielinski et al. 2014). While *BMP4* and *OTX2* are good candidates, we stress that further work is required to identify which specific genes affect variation in bill length. Further, bill length is almost certainly a polygenic trait (e.g., Abzhanov et al. 2006; Lamichhaney et al. 2015, 2016), and many causative loci have likely gone undetected in this study.

There are now several examples linking variation at avian bills, at both the genetic and phenotypic levels, which have shown natural selection to be the primary driver of bill shape variation (Lamichhaney et al. 2015; Bosse et al. 2017). Here, we revealed a striking relationship between genetic variation at our candidate bill‐length SNP 8914s1, and population‐level variation in bill length; the long‐bill allele identified using birds from the Canary Islands is at near fixation in the Madeiran Islands, where birds have the longest bills (Fig. 4C). We then found that variation at SNP 8914s1 was correlated with genome‐wide variation among populations (Fig. S5) with the lowest levels of diversity in the bottlenecked Madeiran and Selvagens populations (Fig. 4C). Combined with the finding that SNP 8914s1 was not identified in the EigenGWAS analysis, this suggests that founder effects are likely to have played a major role in shaping bill length variation among Berthelot's pipit populations. This is consistent with our previous phenotypic work on this species (Spurgin et al. 2014), but only now have we been able to show how founder effects simultaneously affect phenotypes and underlying genotypes—indeed, very few studies have done so.

Despite a clear role for founder effects in shaping bill length variation, we did find that genetic structuring at SNP 8914s1 was higher than the genome‐wide average, particularly across the Canary Islands and Madeira archipelagos (Fig. S5). We also detected an EigenGWAS outlier SNP (8322s2) putatively under divergent selection within the wider *BMP4*/*OTX2* bill‐length candidate region (Fig. S9). Furthermore, we might not expect to be able to identify genotype–phenotype associations at SNPs under strongest divergent selection, as these are expected to have low variability within populations (indeed this is the case in our data—Fig. S10) and detecting rare genetic variants associated with phenotypes is very difficult (Li and Leal 2008). Thus, while the majority of observed bill length variation among archipelagos can be explained by neutral forces, we cannot rule out that selection has occurred for longer bills in Madeira (or shorter bills in southerly populations). Further research is required to identify whether selection has played any role in shaping beak morphology, and the underlying genetic variants associated with this key ecological trait.

Malaria can act as a strong selective force (Warner 1968; Van Riper et al. 1986; Ortego et al. 2008; Knowles et al. 2010), and drive the evolution of increased tolerance to infection (Atkinson et al. 2013). Genotypic associations with malaria infection in individual Berthelot's pipits revealed two outlier SNPs with high levels of significance (Table 2). The first of these was on chromosome 10, approximately 2000 bp from *IL‐16*. This gene encodes a proinflammatory cytokine that has been implicated in susceptibility to various inflammatory disorders (Gao et al. 2009; Wu et al. 2011), and interacts with other cytokines that are associated with malaria infection (Kern et al. 1989; Mathy et al. 2000; Lyke et al. 2004). The second outlier SNP was found on chromosome 20, within *RIMS4*. Research on this gene is lacking, though it has been found to be overexpressed in breast cancer tumor cells (Abba et al. 2005).

Examining allele frequency variation at the malaria resistance candidate SNPs revealed a complex set of relationships. Despite finding a clear relationship of malaria infection with both individual‐ and population‐level SNP variation in the Canary Islands, this pattern did not hold when the Madeiran and Selvagens archipelagos were included (Fig. 3). Further, our EigenGWAS analysis did not detect signatures of divergent selection between archipelagos in the regions surrounding the malaria candidates *RIMS4* or *IL‐16*. In contrast to bill length, which seems more likely to have a conserved genetic architecture, resistance to malaria is probably a rapidly evolving trait, and candidate‐gene studies have shown that different alleles can be associated with malaria infection among different populations (Bonneaud et al. 2006). Variation at both malaria candidate SNPs was correlated with genome‐wide variation (Fig. S5) and likely to be shaped by founder effects. However, our study suggests that, because the genetic basis of malaria resistance is likely to vary among populations (as expected with rapid host–pathogen coevolution, Slade and McCallum 1992), there is unlikely to be a simple relationship between resistance to malaria infection and population demography in wild populations.

The EigenGWAS analysis detected putative signatures of selection among the archipelagos at a number of locations around the genome (Table 3). The significant EigenGWAS SNPs were all in high LD with one another (Fig. S11), suggesting that these sites have evolved nonindependently. Also important to note is that many of these SNPs are not situated close to candidate genes. Some of these could situated close to features such as trans‐regulatory elements or structural variants (Yvert et al. 2003; Manolio et al. 2009), or could be false positives that have arisen as a result of population bottlenecks (Akey et al. 2004). Our analyses suggest that the EigenGWAS analysis was more conservative than a simple *F*_ST_‐based approach to detecting selection, with numerous *F*_ST_ outliers having nonsignificant EigenGWAS *P* values after employing a genomic control (Fig. S8). This is in contrast to other studies that have shown almost perfect correlations between *F*_ST_ and EigenGWAS *P* values (Chen et al. 2016; Bosse et al. 2017), and suggests that EigenGWAS may be more conservative when levels of drift are high. However, it is unlikely that all false positives are accounted for, and further research is now needed to understand how EigenGWAS performs in bottlenecked populations.

Confounding effects of bottlenecks aside, a substantial number of the SNPs identified in our EigenGWAS analyses are likely to be “true” positives, suggesting that traits other than bill length and malaria resistance may have been under selection among archipelagos. We identified SNPs located within loci involved in immune function (e.g., *PREX1*, Welch et al. 2005; *MAP3K14*, Thu and Richmond 2010), or with potential to enable adaptation to climate (e.g., *DLAT*, Blier and Guderley 1993; *SNX29*, Sung et al. 2016). These findings are reflected in other studies that utilize genome scans to detect signatures of selection. Adaptation to toxic food sources in an isolated population of *Drosophila yakuba* was detected in a genome scan between island and mainland populations (Yassin et al. 2016). Divergent selection for pathogen‐resistance candidate genes has been found in *Daphnia* (Bourgeois et al. 2017) and across a bank vole range expansion (White et al. 2013), and pathogens were the strongest selective pressure identified in human evolutionary history (Fumagalli et al. 2011). Likewise, signatures of divergent selection have been detected for genes related to metabolic processes along latitudinal gradients in numerous species (Sezgin et al. 2004; Hancock et al. 2011; Pujolar et al. 2014). This suggests that there may be general patterns in the types of genes that show divergence between populations, with similar processes of adaptation across species. Future studies investigating links between genotypes under selection and ecologically important phenotypic traits will provide further support for the role of adaptation in driving patterns of biodiversity.

Associate Editor: Z. Gompert

## Supporting information


**Table S1**. CEGMA and BUSCO results for the Berthelot's pipit genome assembly.
**Table S2**. Nucleotide diversity across groups of Berthelot's pipits and tawny pipits.
**Table S3**. Outlier SNPs with PIP > 0.1 in BSLMM analyses for bill length and malaria in the "Berthelot's" dataset
**Table S4**. Outlier SNPs from Eigenvector 2 of EigenGWAS analysis.
**Figure S1**. Nucleotide diversity in Tawny pipits and Berthelot's pipit archipelagos
**Figure S2**. Cross‐validation (CV) error for *K* = 1 to *K* = 13 clusters, calculated by Admixture analysis of Berthelot's pipit populations
**Figure S3**. Admixture analysis at *K* = 7 to *K* = 13 clusters for Berthelot's pipit
**Figure S4**. Pairwise genetic distance in relation to geographical distance across Berthelot's pipit populations in the Canary Islands
**Figure S5**. The relationship between BSLMM outlier SNP pairwise *F*_ST_ and A) pairwise genome‐wide *F*_ST_; and B) pairwise geographic distance between all pairs of Berthelot's pipit populations
**Figure S6**. Genetic associations with tarsus length.
**Figure S7**. Genetic associations with malaria and bill length in the "Berthelot's" dataset
**Figure S8**. Selection across archipelagos.
**Figure S9**. Region of interest for bill length on chromosome 5
**Figure S10**. Individual observed heterozygosity averaged across SNPs taken from EigenGWAS outliers (candidate regions ‐ see main text) versus the rest of the genome (non‐candidate regions)
**Figure S11**. Linkage disequilibrium between all pairs of outlier SNPs from EigenGWAS Eigenvector 1 and malaria BSLMM analysis
