# Supplementary material for: Genomic associations with bill length and disease reveal drift and selection across island bird populations
Source: Evol Lett. 2018 Jan 26;2(1):22–36. doi: 10.1002/evl3.38 (PMC6121843; doi:10.1002/evl3.38)
Supplement: Supplementary file 1 — Table S1. CEGMA and BUSCO results for the Berthelot's pipit genome assembly. Table S2. Nucleotide diversity across groups of Berthelot's pipits and tawny pipits. Table S3. Outlier SNPs with PIP > 0.1 in BSLMM analyses for bill length and malaria in the "Berthelot's" dataset Table S4. Outlier SNPs from Eigenvector 2 of EigenGWAS analysis. Figure S1. Nucleotide diversity in Tawny pipits and Berthelot's pipit archipelagos Figure S2. Cross‐validation (CV) error for K = 1 to K = 13 clusters, calculated by Admixture analysis of Berthelot's pipit populations Figure S3. Admixture analysis at K = 7 to K = 13 clusters for Berthelot's pipit Figure S4. Pairwise genetic distance in relation to geographical distance across Berthelot's pipit populations in the Canary Islands Figure S5. The relationship between BSLMM outlier SNP pairwise F ST and A) pairwise genome‐wide F ST; and B) pairwise geographic distance between all pairs of Berthelot's pipit populations Figure S6. Genetic associations with tarsus length. Figure S7. Genetic associations with malaria and bill length in the "Berthelot's" dataset Figure S8. Selection across archipelagos. Figure S9. Region of interest for bill length on chromosome 5 Figure S10. Individual observed heterozygosity averaged across SNPs taken from EigenGWAS outliers (candidate regions ‐ see main text) versus the rest of the genome (non‐candidate regions) Figure S11. Linkage disequilibrium between all pairs of outlier SNPs from EigenGWAS Eigenvector 1 and malaria BSLMM analysis [file EVL3-2-22-s001.docx]

### Genomic associations with bill length and disease reveal drift and selection across island bird populations

**Supplementary Material**

Supplementary Methods, Supplementary Results, Supplementary Tables and Figures

**Authors:**

Claire Armstrong^1^, David S. Richardson^1^*, Helen Hipperson^2^, Gavin J. Horsburgh^2^, Clemens Küpper^3^, Lawrence Percival-Alwyn^4^, Matt Clark^4^, Terry Burke^2^ and Lewis G. Spurgin^1^*

*Correspondence: [David.Richardson@uea.ac.uk](mailto:David.Richardson@uea.ac.uk); [L.Spurgin@uea.ac.uk](mailto:L.Spurgin@uea.ac.uk)

1. School of Biological Sciences, University of East Anglia, Norwich Research Park, Norwich, NR4 7TJ, United Kingdom
2. NERC Biomolecular Analysis Facility, Department of Animal and Plant Sciences, University of Sheffield, S10 2TN, United Kingdom
3. Max Planck Institute for Ornithology, Eberhard-Gwinner-Str., 82319 Seewiesen, Germany
4. Earlham Institute, Norwich Research Park, Norwich, NR4 7UZ, United Kingdom

### Supplementary Methods

##### Sample collection

Birds were caught in spring traps baited with *Tenebrio molitor* larvae, and each bird was fitted with either a unique numbered aluminium ring issued from the Spanish or Portuguese ministries, or with a coloured plastic ring. Blood samples were obtained by brachial venipuncture and stored at room temperature in 800 μl of 100% ethanol in screw-cap microcentrifuge tubes. Bill length and tarsus length were measured with callipers to the nearest 0.1 mm. The age of the birds was determined as adult or juvenile based on feather moult pattern (Cramp 1988). Further information on each bird was derived from the blood samples as detailed in Illera *et al.* (2008) and Spurgin *et al.* (2012). In brief, DNA was extracted using a salt extraction protocol described in Richardson *et al.* (2001), and molecularly sexed (Griffiths *et al.* 1998). Haemosporidian blood parasites such as *Plasmodium* were detected using a nested PCR approach (Waldenström *et al.* 2004), and PCR products from samples with infections were Sanger-sequenced to characterise the strains present across Berthelot's pipit populations.

##### Berthelot's pipit genome

A draft Berthelot's pipit genome was prepared using a sample collected from Porto Santo, Madeira. This bird was selected due to its low level of heterozygosity, calculated from the RAD data (see below). The genome was used in this study to increase the number of RAD loci successfully mapped to the zebra finch genome.

Sample preparation, sequencing, and genome assembly were performed at the Earlham Institute in Norwich. 10 μg of blood was extracted with the Agencourt Genfind V2 Blood & Serum Genomic DNA Isolation Kit (Beckman Coulter). Following extraction, DNA concentration was measured using the Qubit dsDNA HS Assay Kit (Life Technologies). Five hundred nanograms of genomic DNA was sheared into ~400 bp fragments using a Covaris S2 acoustic sonicator (Covaris Inc.), with the following shearing parameters: duty cycle = 10%, intensity = 5, cycles per burst = 200, time = 45 seconds. DNA fragments were then cleaned using 0.6x AMPure XP beads (Beckman Coulter) and eluted into 40 ul Tris-HCl pH 8.0. A further cleaning with 3.0x AMPure XP beads without elution was followed by end repair, A-tailing and ligation of Illumina PCR-free TruSeq adapters (Illumina) using KAPA Library Preparation Kit reagents (Kapa Biosystems). An additional 0.7x AMPure XP bead clean up removed adapter dimers and fragments shorter than ~150 bp. The library was then eluted off the AMPure beads. The library was QC checked with the Bioanalyzer DNA HS assay system (Agilent Technologies Inc.) and quantified by both Qubit dsDNA HS Assay Kit and qPCR using a KAPA Library Quantification Kit on a StepOnePlus Real-Time PCR System (Life Technologies). Illumina sequencing was performed using paired-end sequencing (2 x 125 bp), with a 1% PhiX spike, on an Illumina HiSeq 2500 sequencer in rapid-run mode. Following sequencing, read quality was assessed with FastQC (www.bioinformatics.babraham.ac.uk/projects/fastqc/). The draft genome was assembled using DISCOVAR *de novo* (Weisenfeld *et al.* 2014), and assembly statistics were calculated with the "abyss-fac" utility in ABySS (Simpson *et al.* 2009). The completeness of the genome was assessed using CEGMA (Parra *et al.* 2007) and BUSCO (Simão *et al.* 2015), which searched the genome for 248 highly conserved core eukaryotic genes and 3023 vertebrate-specific single copy orthologs, respectively.

##### RAD library preparation

DNA was extracted from samples using a salt extraction protocol (Richardson *et al.* 2001), using a sufficient quantity of blood to gain a DNA concentration >50 ng/µl. The quality of the DNA extractions was visually assessed under UV after electrophoresis on a 1.2% agarose gel, and DNA concentration was quantified with Quantifluor dsDNA dye (Promega) on a FLUOstar OPTIMA microplate reader (BMG Labtech Ltd). Library preparation was carried out at the NERC Biomolecular Analysis Facility in Sheffield, following a modified version of the ddRAD protocol by DaCosta & Sorenson (2014). Samples were allocated to one of three libraries, with populations evenly spread across the libraries to reduce bias from library-specific effects. 1 μg of DNA was digested with high fidelity SbfI and EcoRI restriction enzymes (New England Biolabs). P1 and P2 sequencing adapters were ligated onto the digested fragments. These adapters include the Illumina TruSeq amplification and sequencing primer sequences, a unique eight bp barcode sequence, and overhangs to ligate to the sticky ends produced by SbfI and EcoRI, respectively (details of primer design in DaCosta & Sorenson 2014). The DNA concentration of each sample was then quantified on a StepOnePlus Real-Time PCR System (Applied Biosystems), and samples were pooled into 12 pools of equimolar amounts. Pools were size-selected for fragments between 300 and 450 bp by cutting from a 2% low melt agarose gel with size standards added to each pool, and purified using a MinElute Gel Extraction Kit (Qiagen). Pools were PCR amplified with Phusion High-Fidelity PCR Master Mix (Finnzymes) and purified with AMPure XP beads (Agencourt), then quantified as above. The 12 pools were then mixed in equimolar amounts into a final pool of at least 2 nM concentration, which was sent to Edinburgh Genomics for sequencing on an Illumina HiSeq2500.

##### RAD library bioinformatics

The raw RAD sequencing data was first quality-checked using FastQC (www.bioinformatics.babraham.ac.uk/projects/fastqc/), then processed using the ddRAD pipeline created by DaCosta & Sorenson (2014). This pipeline first demultiplexes the reads, assigning them to samples based on the 8 bp barcode sequence. The following was performed on each dataset independently. Reads were sorted by quality and clustered with UCLUST in USEARCH version 5 (Edgar 2010) at an identity of 85%. In each cluster, the sequence with the highest quality score was then mapped to the zebra finch genome (*Taeniopygia guttata,* version 3.2.4; Warren *et al.* 2010) with BLASTN version 2.2.31 (Altschul *et al.* 1990), and clusters sharing a single BLAST hit were combined. The reads in each cluster were aligned in MUSCLE version 3.8.31 (Edgar 2004). Genotypes were scored using the script *RADGenotypes.py* (see DaCosta & Sorenson 2014 for full details). The "All Pipits" dataset containing all Berthelot's pipit and tawny pipit samples was then filtered to contain all SNPs from RAD loci that were successfully genotyped in 100% of individuals, removing loci that contained SNPs with >2 alleles. Filtering for the other four datasets accepted loci that could not be confidently genotyped in ≤3 samples (these ambiguous genotypes were then treated as missing data in downstream analysis), and allowed up to 10% of samples with missing genotypes. Allele frequencies were calculated with the R package *adegenet* (Jombart 2008), and biallelic SNPs with a minor allele frequency ≥3% were retained. Further filtering resulted in datasets containing one SNP, per RAD locus, that had the highest minor allele frequency within that locus.

The draft Berthelot's pipit genome was used to improve mapping ability for loci across the four Berthelot's pipit datasets that failed to be conclusively mapped to the zebra finch genome. Where possible, the sequences for unmapped loci were taken from the same individual that was used to construct the genome, otherwise the consensus sequence was used. Unmapped sequences were then searched for in the pipit genome using BLASTN version 2.6.0 (Altschul *et al.* 1990). When a single hit to the pipit genome was obtained, the region of the genome spanning 1000 bp in each direction from the RAD locus was recorded. This extended sequence was mapped against the zebra finch genome, using the *runBLAST.py* script from the ddRAD pipeline (DaCosta & Sorenson 2014) and BLASTN version 2.3.0 (Altschul *et al.* 1990). Positions of loci successfully mapped in this way were updated.

A final filtering step was applied to the "Berthelot's" dataset to make a sixth dataset, "Berthelot's HWE" (Table 1) that removes SNPs that could have confounding effects on estimates of population structure. First we removed SNPs located on the Z chromosome, which is evolving under different selective forces to the autosomes (Li & Merilä 2010). We then calculated *p* values for deviations from Hardy-Weinberg Equilibrium (HWE) in PLINK 1.9 (Chang *et al.* 2015), and removed SNPs that were out of HWE at *p* < 0.05 in three or more populations.

##### Associations between BSLMM and EigenGWAS outlier SNPS

We found an apparent overlap between the most significant SNP identified in the BSLMM for bill length, and an outlier SNP identified in the EigenGWAS analysis of EV1 (see main text, Fig. 4). To test whether this overlap was significant, we used a randomisation approach. We created 1000 randomised datasets, in which we randomly selected a SNP, and we calculated the distance between the randomly selected SNP and the closest, genome-wide significant EigenGWAS SNP. We repeated this process 1000 times, and therefore generated a distribution of distances. We compared the distance of our significant BSLMM SNP and the closest EigenGWAS SNP against this distribution of distances in order to generate a one-tailed *p* value.

### Supplementary Results and Discussion

##### Berthelot's pipit genome

The pipit genome sequencing run produced 143,415,538 paired reads, which were assembled in DISCOVAR *de novo* (Weisenfeld *et al.* 2014) to create a draft genome of 1,153,192,274 bp, comprising 350,587 contigs, with a contig N50 of 355,835 bp. The completeness of the genome, assessed through CEGMA (Parra *et al.* 2007) and BUSCO (Simão *et al.* 2015), is given in Table S1.

##### BSLMM analysis in "Berthelot's" dataset

The BSLMM analyses outlined in the Methods were repeated on the "Berthelot's" dataset, to test for additional power in detecting SNPs associated with bill length and malaria infection with a larger sample size. For bill length, a median of 77.6% of phenotypic variation was explained by the genotype (95% CI 55.3–98.2%), of which 14.8% was explained by SNPs of non-zero effects (95% CI 0.0–91.4%). Associations with malaria infection found a median of 84.0% of variation in phenotype explained by genotype (95% CI 43.2–99.9%), with 23.8% of this explained by SNPs of non-zero effects (95% CI 0.0–84.8%). Outlier SNPs with a posterior inclusion probability > 0.1 are listed in Table S3.

*PCR duplicates*

Any analysis that requires high genotyping accuracy at individual loci, such as our BSLMM and EigenGWAS analyses, are susceptible to erroneous results due to PCR duplicates (Andrews *et al.* 2016). However, in our case PCR duplicates are likely to make our analyses conservative. For the BSLMM analyses, PCR duplicates will be random with respect to bill length, so if anything will obscure relationships between genotype and bill length. A greater possibility is that PCR duplicates may generate false signatures of divergent selection. However, the high levels of LD between all of our candidate SNPs from the EigenGWAS analysis suggest a highly correlated, non-random pattern of evolution. It is therefore unlikely that PCR duplicates have introduced a systematic bias into our results.

### References

Altschul, S.F., Gish, W., Miller, W., Myers, E.W. & Lipman, D.J. (1990). Basic local alignment search tool. *J. Mol. Biol.*, 215, 403–410.

Andrews, K. R., Good, J. M., Miller, M. R., Luikart, G., & Hohenlohe, P. A. (2016). Harnessing the power of RADseq for ecological and evolutionary genomics. *Nat. Rev. Gen.,* 17, 81-92.

Chang, C.C., Chow, C.C., Tellier, L.C., Vattikuti, S., Purcell, S.M. & Lee, J.J. (2015). Second-generation PLINK: rising to the challenge of larger and richer datasets. *Gigascience*, 4, 7.

Cramp, S. (1988). *The Birds of the Western Palearctic, Volume 5*. Oxford University Press, Oxford, UK.

DaCosta, J.M. & Sorenson, M.D. (2014). Amplification biases and consistent recovery of loci in a double-digest RAD-seq protocol. *PLoS One*, 9, e106713.

Edgar, R.C. (2004). MUSCLE: Multiple sequence alignment with high accuracy and high throughput. *Nucleic Acids Res.*, 32, 1792–1797.

Edgar, R.C. (2010). Search and clustering orders of magnitude faster than BLAST. *Bioinformatics*, 26, 2460–2461.

Griffiths, R., Double, M.C., Orr, K. & Dawson, R.J.G. (1998). A DNA test to sex most birds. *Mol. Ecol.*, 7, 1071–1075.

Illera, J.C., Emerson, B.C. & Richardson, D.S. (2008). Genetic characterization, distribution and prevalence of avian pox and avian malaria in the Berthelot’s pipit (*Anthus berthelotii*) in Macaronesia. *Parasitol. Res.*, 103, 1435–1443.

Jombart, T. (2008). Adegenet: a R package for the multivariate analysis of genetic markers. *Bioinformatics*, 24, 1403–1405.

Li, M.-H. & Merilä, J. (2010). Sex-specific population structure, natural selection, and linkage disequilibrium in a wild bird population as revealed by genome-wide microsatellite analyses. *BMC Evol. Biol.*, 10, 66.

Parra, G., Bradnam, K. & Korf, I. (2007). CEGMA: a pipeline to accurately annotate core genes in eukaryotic genomes. *Bioinformatics*, 23, 1061–1067.

Richardson, D.S., Jury, F.L., Blaakmeer, K., Komdeur, J. & Burke, T. (2001). Parentage assignment and extra-group paternity in a cooperative breeder: The Seychelles warbler (*Acrocephalus sechellensis*). *Mol. Ecol.*, 10, 2263–2273.

Simão, F.A., Waterhouse, R.M., Ioannidis, P., Kriventseva, E.V. & Zdobnov, E.M. (2015). BUSCO: assessing genome assembly and annotation completeness with single-copy orthologs. *Bioinformatics*, 31, 3210–3212.

Simpson, J.T., Wong, K., Jackman, S.D., Schein, J.E., Jones, S.J. & Birol, İ. (2009). ABySS: A parallel assembler for short read sequence data. *Genome Res.*, 19, 1117–1123.

Spurgin, L.G., Illera, J.C., Padilla, D.P. & Richardson, D.S. (2012). Biogeographical patterns and co-occurrence of pathogenic infection across island populations of Berthelot’s pipit (*Anthus berthelotii*). *Oecologia*, 168, 691–701.

Waldenström, J., Bensch, S., Hasselquist, D. & Östman, Ö. (2004). A new nested polymerase chain reaction method very efficient in detecting *Plasmodium* and *Haemoproteus* infections from avian blood. *J. Parasitol.*, 90, 191–194.

Warren, W.C., Clayton, D.F., Ellegren, H., Arnold, A.P., Hillier, L.W. & Künstner, A. *et al.* (2010). The genome of a songbird. *Nature*, 464, 757–762.

Weisenfeld, N.I., Yin, S., Sharpe, T., Lau, B., Hegarty, R. & Holmes, L. *et al.* (2014). Comprehensive variation discovery in single human genomes. *Nat. Genet.*, 46, 1350–1355.

### Supplementary Tables and Figures

**Table S1** CEGMA and BUSCO results for the Berthelot’s pipit genome assembly.

|  | CEGMA | | BUSCO vertebrate genes | | | |
| --- | --- | --- | --- | --- | --- | --- |
|  | Complete | Partial | Complete single-copy | Complete duplicated | Fragmented | Missing |
| Berthelot's pipit draft genome | 152 (61%) | 224 (90%) | 1944 (64%) | 21 (0.6%) | 476 (15%) | 603 (19%) |

**Table S2** Nucleotide diversity across groups of Berthelot's pipits and tawny pipits. Mean and bootstrapped 95% confidence intervals for π_SNP_ (per-SNP heterozygosity) and π_RAD_ (nucleotide diversity per RAD locus). In addition to archipelago-level grouping in Berthelot's pipit, this analysis was repeated with Madeira split according to population structuring identified in ADMIXTURE.

| Dataset | Mean π_SNP_ | 95% CI π_SNP_ | Mean π_RAD_ | 95% CI π_RAD_ |
| --- | --- | --- | --- | --- |
| Tawny pipits | 0.1043 | 0.1019-0.1066 | 0.00488 | 0.00466-0.00510 |
| Canary Islands | 0.0118 | 0.0107-0.0128 | 0.00053 | 0.00047-0.00059 |
| Madeira | 0.0079 | 0.0069-0.0090 | 0.00036 | 0.00030-0.00043 |
| Madeira excl. DG | 0.0077 | 0.0066-0.0087 | 0.00035 | 0.00029-0.00041 |
| DG | 0.0078 | 0.0067-0.0089 | 0.00036 | 0.00028-0.00042 |
| Selvagens | 0.0058 | 0.0048-0.0067 | 0.00027 | 0.00022-0.00032 |

**Table S3** Outlier SNPs with PIP > 0.1 in BSLMM analyses for bill length and malaria in the "Berthelot's" dataset. The *p* value from the LMM analyses is shown. The nearest gene within 10,000 bp of the SNP is identified.

| Phenotype | SNP | Locus ID | PIP | LMM *p* | Position | Gene | Distance (bp) |
| --- | --- | --- | --- | --- | --- | --- | --- |
| Bill length | 3916s2 | 1990 | 0.40 | 0.0001 | Chr28:3343405 | - | - |
|  | 176s3 | 86 | 0.24 | 0.0007 | Chr21:5714527 | *PLEKHN1* | 4828 |
|  | 4903s3 | 2562 | 0.17 | 0.001 | Chr15:1987982 | - | - |
|  | 1350s3 | 656 | 0.17 | 0.002 | Chr23:1959411 | - | - |
|  | 6668s3 | 3542 | 0.13 | 0.003 | Chr7:38943610 | - | - |
|  | 3384s3 | 1679 | 0.13 | 0.0005 | - | - | - |
|  | 8914s1 | 4494 | 0.12 | 0.001 | Chr5:60950937 | - | - |
|  | 4605s3 | 2390 | 0.12 | 0.003 | Chr4A:18361942 | - | - |
|  | 8588s1 | 4410 | 0.11 | 0.006 | Chr3:35547677 | *FEZ2* | In gene |
|  | 6170s2 | 3269 | 0.10 | 0.005 | Chr2:58237321 | - | - |
| Malaria | 7259s1 | 3870 | 0.49 | 0.00006 | Chr20:6483195 | *RIMS4* | In gene |
|  | 1303s1 | 632 | 0.34 | 0.0002 | Chr1A:50095389 | *CACNA1I* | In gene |
|  | 370s2 | 194 | 0.21 | 0.002 | Chr11:20923524 | *CDH11* | In gene |
|  | 207s3 | 108 | 0.19 | 0.002 | Chr24:2861103 | *TBCEL* | In gene |
|  | 3431s2 | 1710 | 0.18 | 0.004 | Chr21:4658523 | - | - |
|  | 13s1 | 5 | 0.18 | 0.0008 | Chr1:22008821 | *NLGN4X* | In gene |
|  | 5239s1 | 2739 | 0.17 | 0.002 | Chr10:12048280 | *IL-16* | 2188 |
|  | 5812s3 | 3077 | 0.15 | 0.001 | Chr15:1405244 | - | - |
|  | 3159s1 | 1563 | 0.14 | 0.002 | Chr26:88595 | *ALX3* | 6137 |
|  | 8626s5 | 4425 | 0.13 | 0.007 | Chr7:37287107 | - | - |
|  | 3087s3 | 1531 | 0.12 | 0.01 | ChrZ:49829841 | *KIF2A* | In gene |
|  | 8222s3 | 4253 | 0.12 | 0.02 | Chr19:10536989 | *MSI2* | In gene |
|  | 3054s3 | 1514 | 0.11 | 0.01 | - | - | - |
|  | 3135s1 | 1548 | 0.11 | 0.007 | Chr4:62637967 | *ZFYVE28* | In gene |
|  | 565s1 | 283 | 0.10 | 0.007 | Chr5:58806769 | *ATG14* | In gene |
|  | 2824s1 | 1378 | 0.10 | 0.003 | Chr4:47092989 | *SLC30A9* | In gene |
|  | 8827s1 | 4480 | 0.10 | 0.01 | - | - | - |
|  | 6877s4 | 3657 | 0.10 | 0.03 | Chr27:173314 | *UBTF* | 4966 |

**Table S4** Outlier SNPs from Eigenvector 2 of EigenGWAS analysis. The nearest gene within 10,000 bp of the SNP is identified.

| SNP | Locus ID | *p* | Position | Gene | Distance (bp) |
| --- | --- | --- | --- | --- | --- |
| 203s1 | 105 | 6 x 10^-26^ | Chr27:1229392 | *MAP3K14* | In gene |
| 7826s2 | 4142 | 2 x 10^-13^ | Chr24:1583716 | *DLAT* | In gene |
| 7622s1 | 4065 | 1 x 10^-11^ | Chr27:1534267 | *GPATCH8* | 965 |
| 5483s1 | 2876 | 4 x 10^-11^ | - | - | - |
| 8418s1 | 4348 | 1 x 10^-10^ | Chr14:7479033 | *SNX29* | In gene |
| 2982s2 | 1469 | 2 x 10^-10^ | - | - | - |
| 2041s1 | 997 | 1 x 10^-9^ | - | - | - |
| 174s1 | 85 | 2 x 10^-8^ | Chr27:1376142 | *MYO1D* | In gene |
| 5752s1 | 3039 | 3 x 10^-8^ | Chr3:105320550 | - | - |
| 8322s2 | 4296 | 5 x 10^-8^ | Chr5:58018667 | *AP5M1* | 2868 |
| 7216s3 | 3844 | 7 x 10^-8^ | - | - | - |
| 4131s1 | 2115 | 9 x 10^-8^ | Chr20:15447166 | *PREX1* | In gene |
| 2473s1 | 1217 | 1 x 10^-7^ | Chr20:15293405 | *CSE1L* | In gene |
| 6932s2 | 3689 | 1 x 10^-7^ | Chr3:96985953 | - | - |
| 7180s1 | 3825 | 2 x 10^-7^ | Chr4A:5912527 | - | - |
| 1728s2 | 849 | 6 x 10^-7^ | - | - | - |
| 6765s1 | 3602 | 7 x 10^-7^ | Chr4A:6655419 | *VSIG4* | In gene |
| 4138s2 | 2120 | 2 x 10^-6^ | Chr20:3241763 | *GSS* | 4 |
| 1723s1 | 846 | 2 x 10^-6^ | Chr12:3914968 | - | - |
| 1541s1 | 760 | 6 x 10^-6^ | Chr2:25304006 | *VPS50* | In gene |
| 2273s1 | 1117 | 1 x 10^-5^ | Chr1A:56816580 | - | - |

**
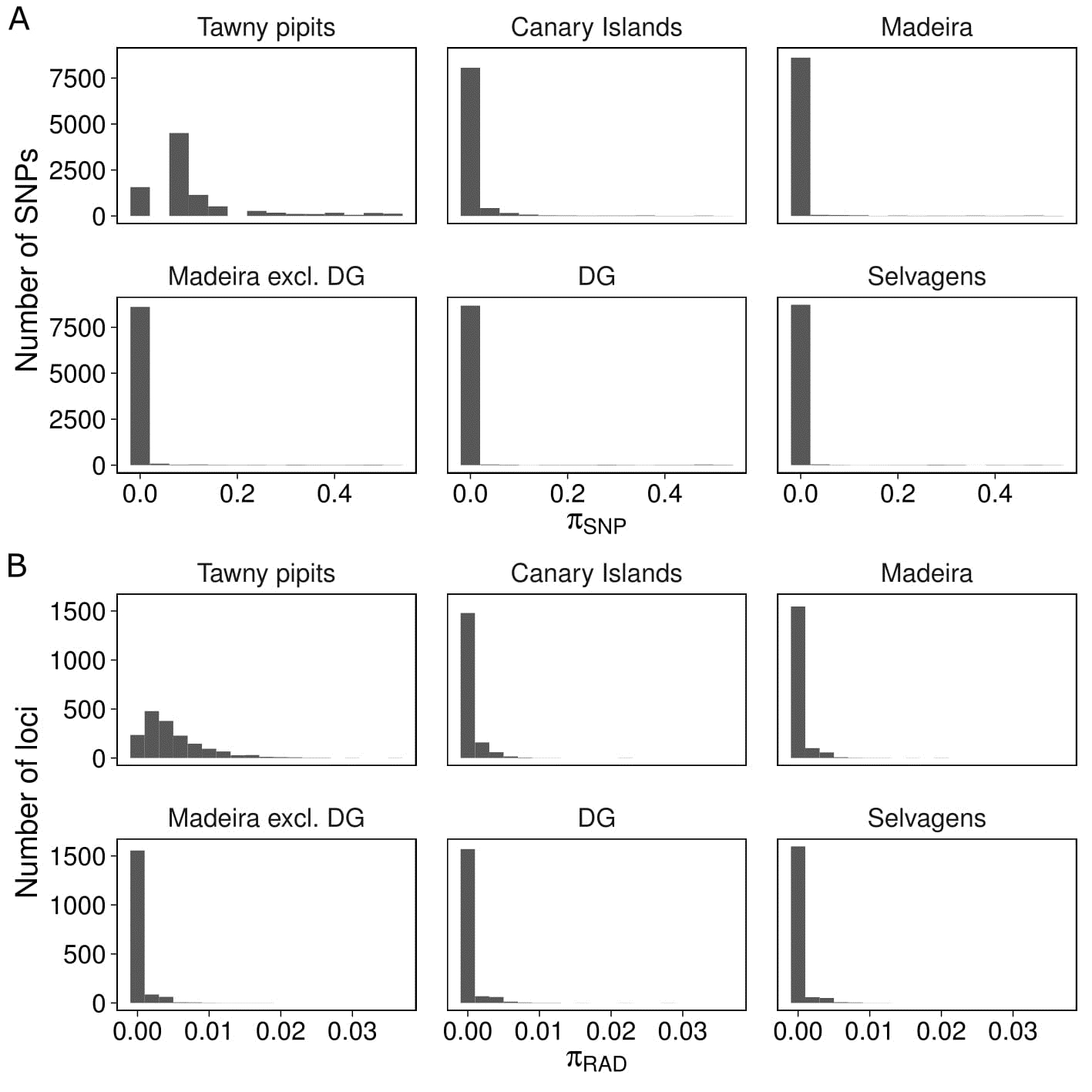
Figure S1** Nucleotide diversity in Tawny pipits and Berthelot's pipit archipelagos. Histograms showing the distributions of A) π_SNP_ and B) π_RAD_ in each group. As Admixture analysis demonstrated moderate population structuring within the Madeiran archipelago (Fig. 2), π_SNP_ and π_RAD_ have been calculated for all islands in the Madeiran archipelago, and additionally separating the more divergent population, Deserta Grande (DG), from Madeira and Porto Santo.

**
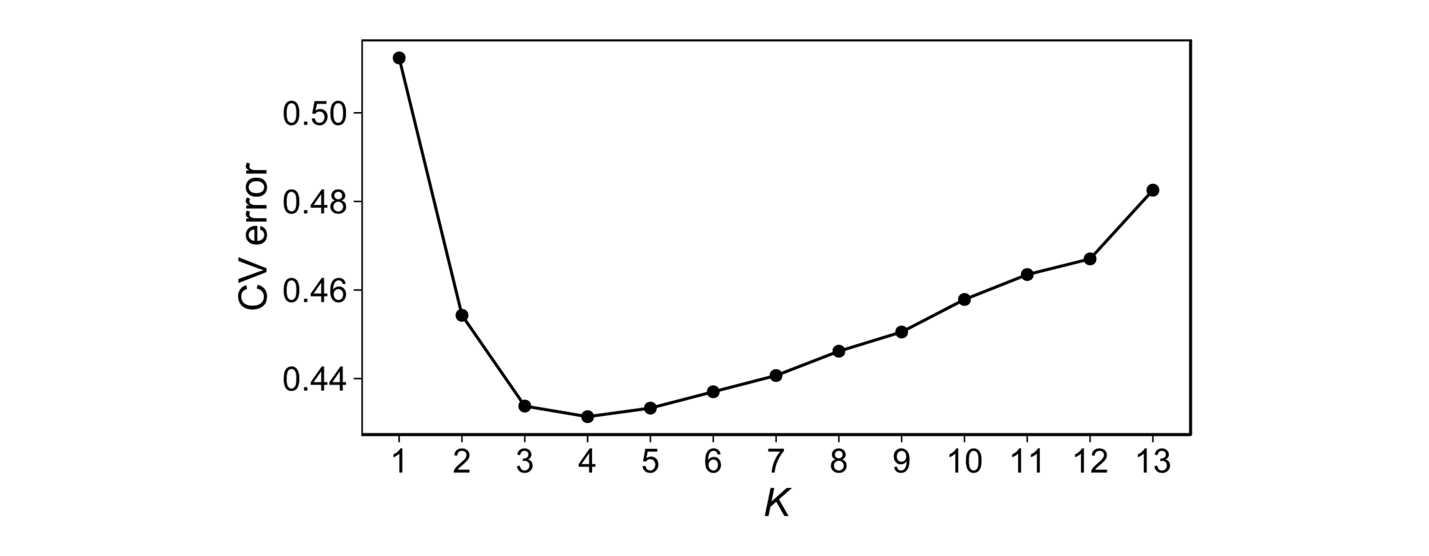
Figure S2** Cross-validation (CV) error for *K* = 1 to *K* = 13 clusters, calculated by Admixture analysis of Berthelot's pipit populations.


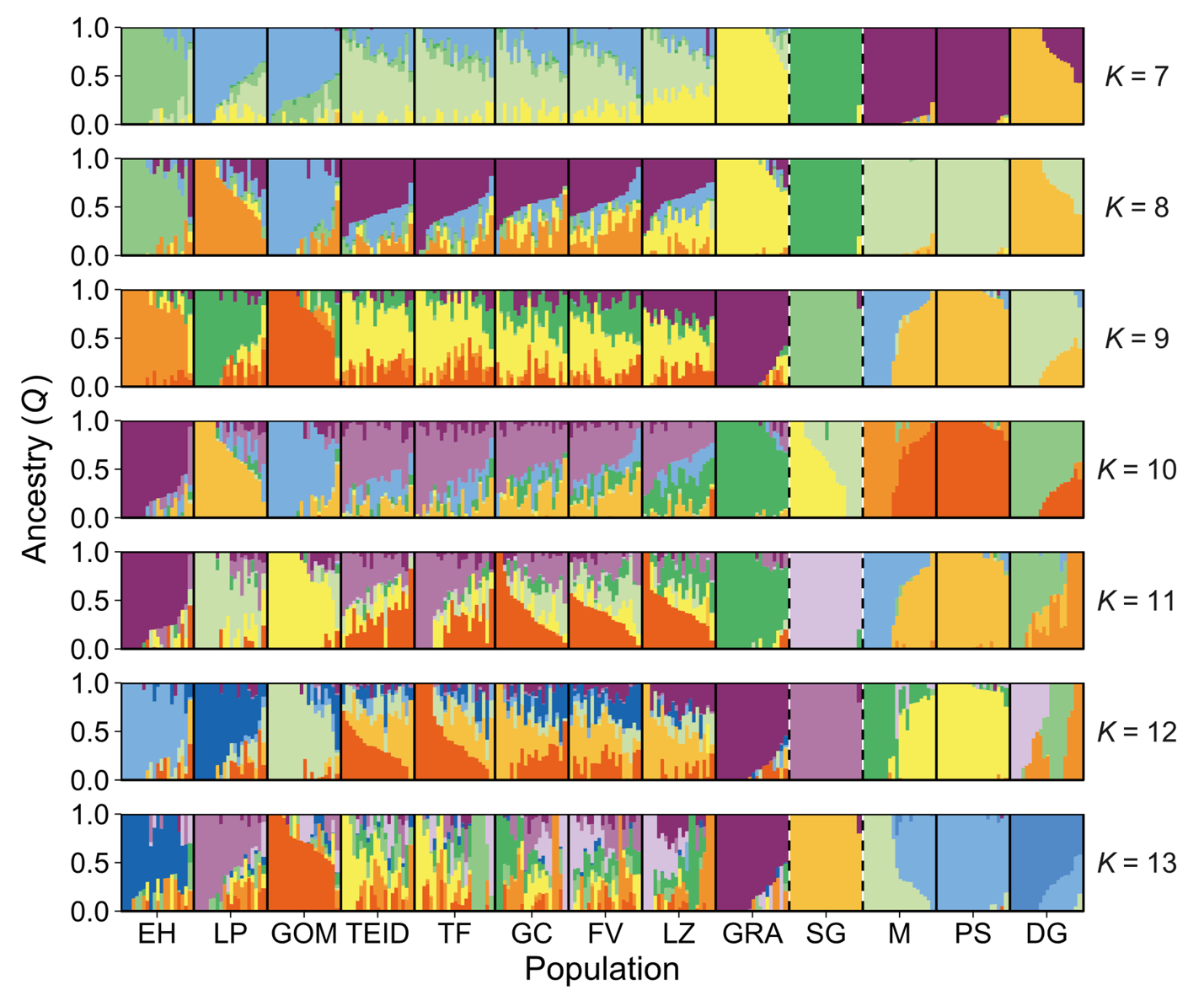
**Figure S3** Admixture analysis at *K* = 7 to *K* = 13 clusters for Berthelot's pipit. Vertical bars represent individual pipits, and are coloured by their assignment to each one of *K* clusters. Solid black lines indicate separate populations; dashed black lines indicate separate archipelagos. Populations from left to right: Canary Islands – El Hierro (EH), La Palma (LP), Gomera (GOM), Teide (TEID), Tenerife (TF), Gran Canaria (GC), Fuerteventura (FV), Lanzarote (LZ), Graciosa (GRA); Selvagens – Selvagem Grande (SG); Madeira – Madeira (M), Porto Santo (PS), Deserta Grande (DG).

**
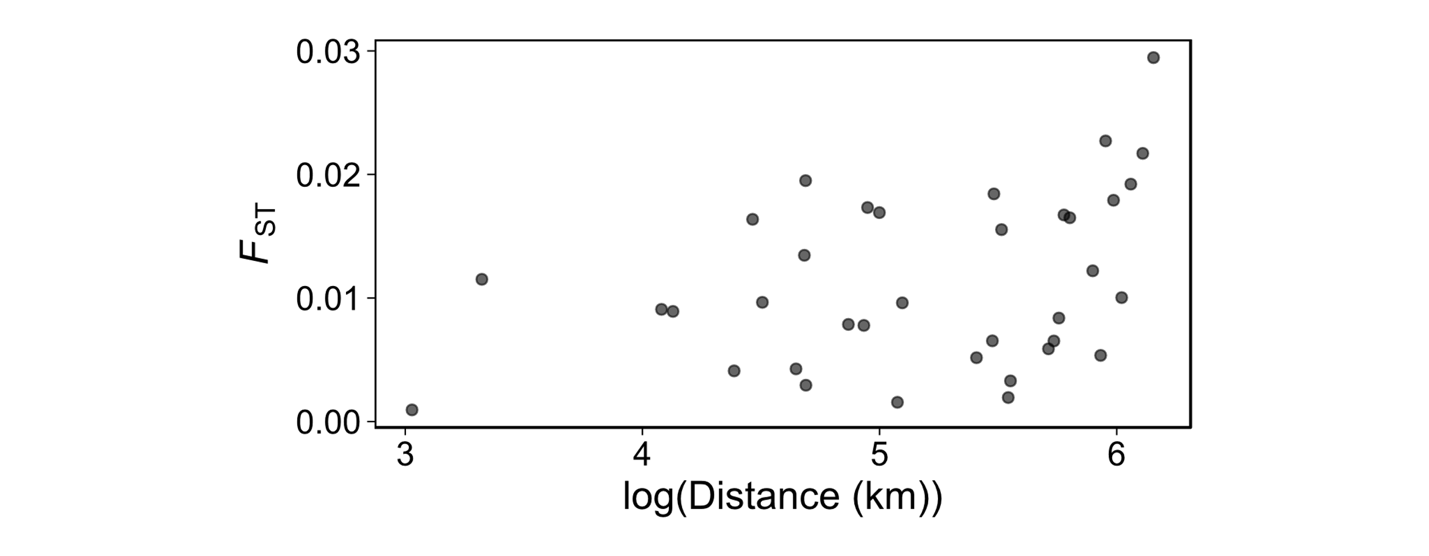
Figure S4** Pairwise genetic distance in relation to geographical distance across Berthelot's pipit populations in the Canary Islands.

**
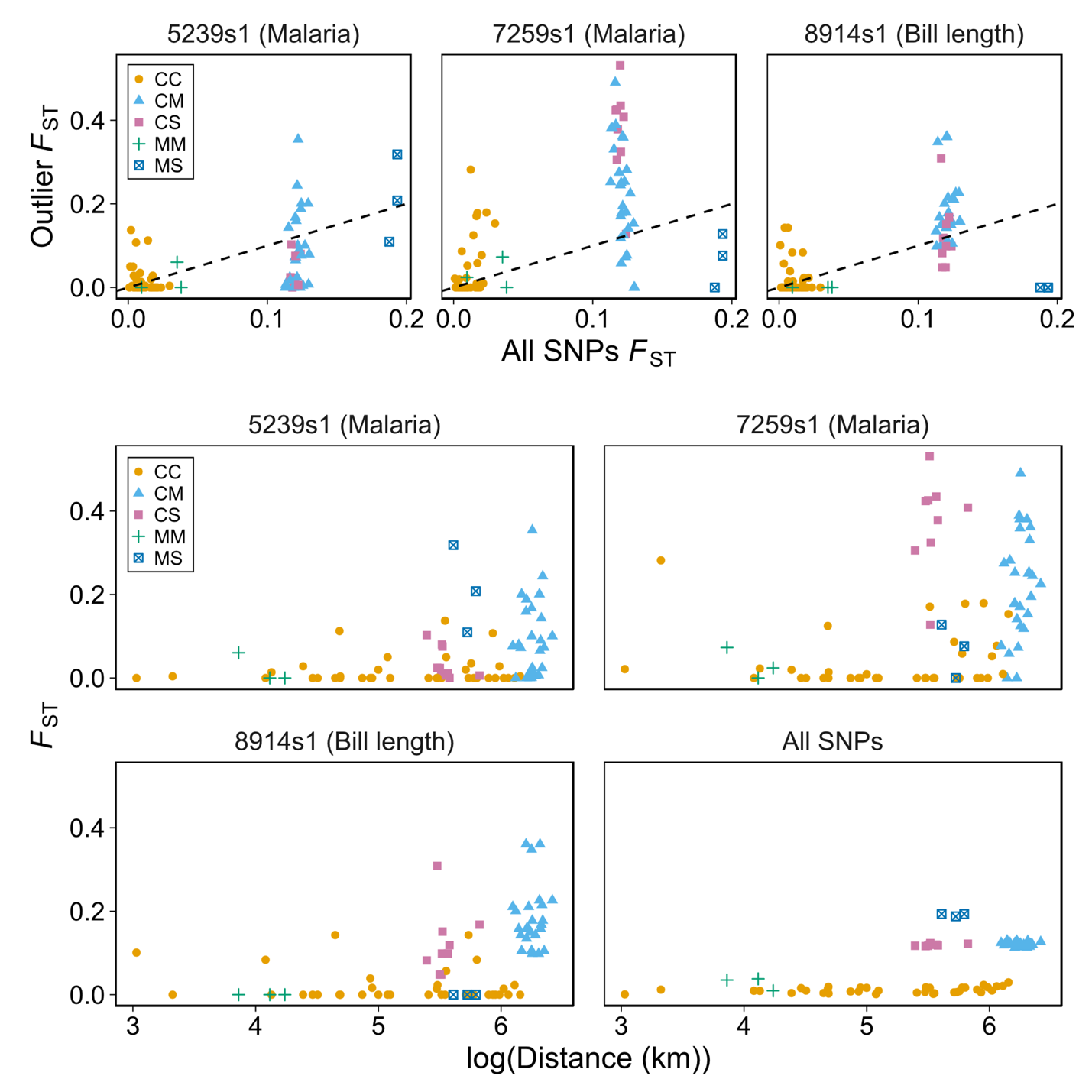
Figure S5** The relationship between BSLMM outlier SNP pairwise *F*_ST_ and A) pairwise genome-wide *F*_ST_; and B) pairwise geographic distance between all pairs of Berthelot's pipit populations. Point colours and symbols represent pairwise comparisons between the different archipelagos, eg. CC = Canary Islands vs. Canary Islands, CM = Canary Islands vs. Madeira, etc. Dashed line in A) indicates a 1:1 relationship.

**
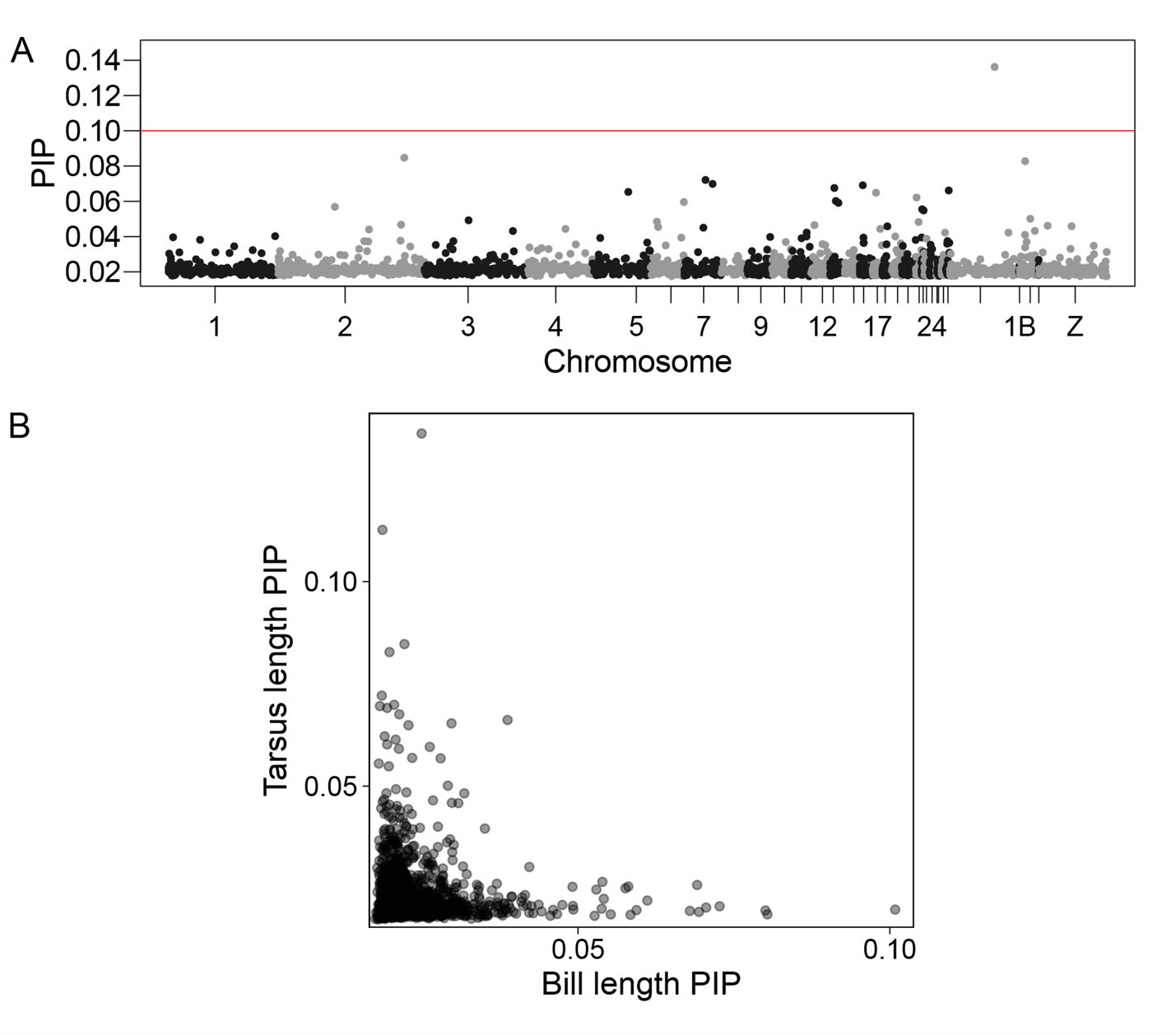
Figure S6** Genetic associations with tarsus length. A) Manhattan plot of BSLMM analysis of tarsus length in the Canary Islands. Red line indicates the threshold of posterior inclusion probability (PIP) = 0.1. B) Tarsus length PIP values plotted against bill length PIP values demonstrate a lack of overlap between outlier SNPs.

**
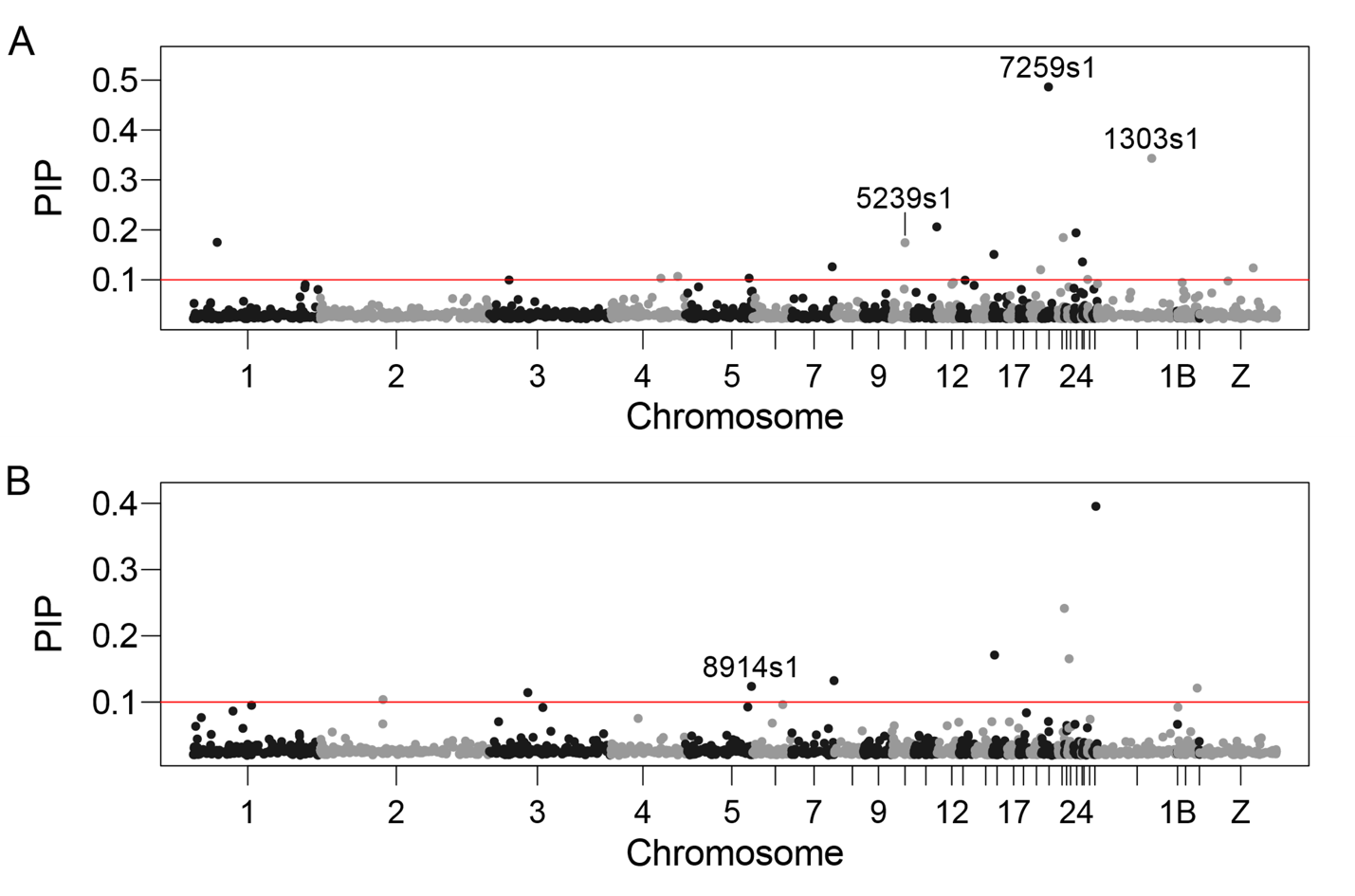
Figure S7** Genetic associations with malaria and bill length in the "Berthelot's" dataset. Manhattan plots of BSLMM analyses for A) malaria infection and B) bill length across all Berthelot's pipit populations. Red line indicates the threshold of posterior inclusion probability (PIP) = 0.1. SNPs with PIPs > 0.1 in both the “Canary Islands” and “Berthelot’s” datasets BSLMM analyses are labelled.

**
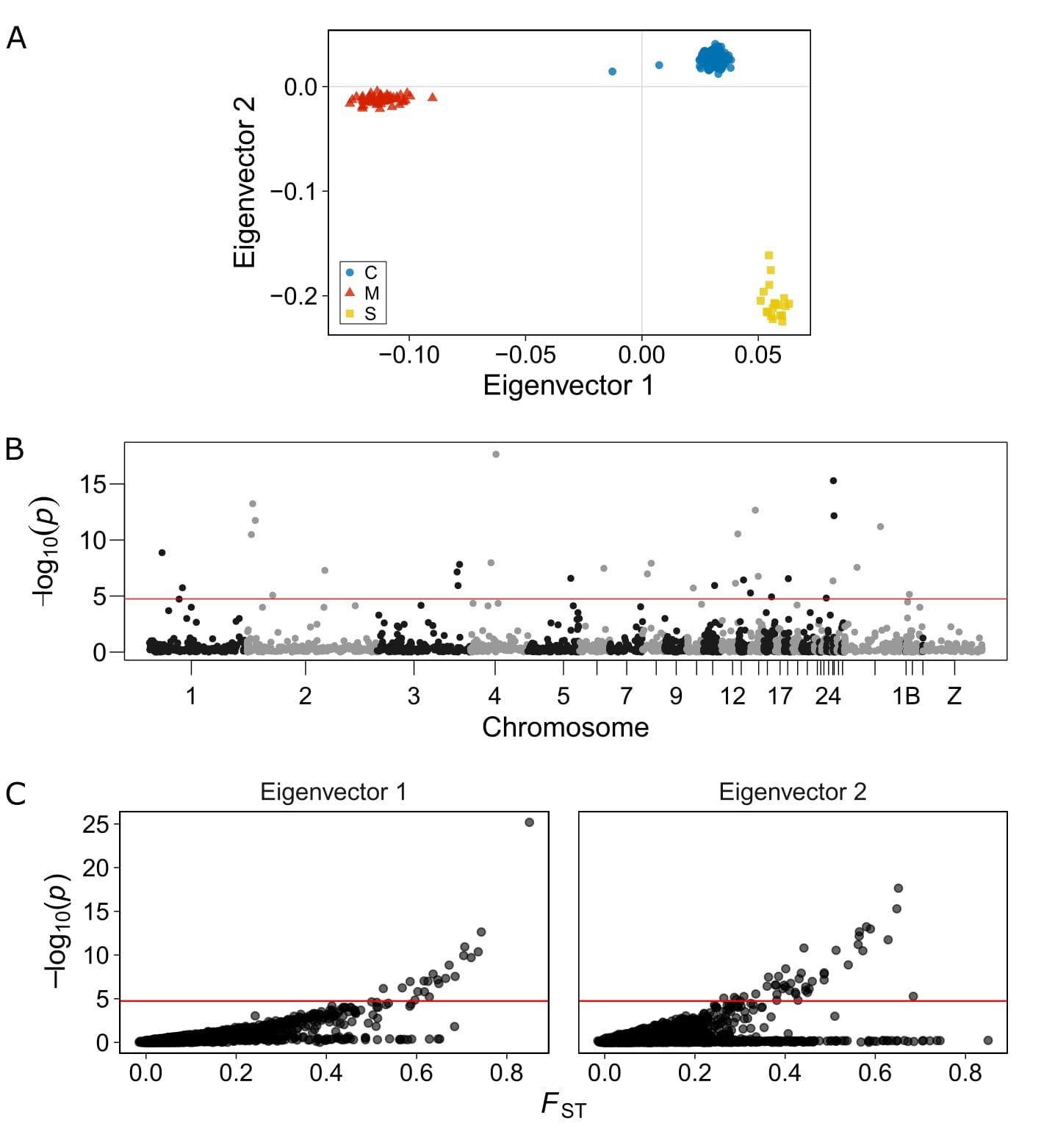
Figure S8** Selection across archipelagos. A) EigenGWAS eigenvalues along eigenvectors (EV) 1 and 2. Points represent individual samples, coloured by archipelago. B) Manhattan plot of EigenGWAS EV2. Red line indicates the Bonferroni-corrected *p* value threshold. C) The relationship between EigenGWAS *p* value and *F*_ST_. In the left-hand plot, per-SNP *F*_ST_ was calculated by grouping individuals according to clustering along Eigenvector 1 (Canary Islands and Selvagens vs. Madeira), and plotted against each SNP's respective EigenGWAS *p* values. Likewise, the right-hand plot groups samples in line with Eigenvector 2 clustering: Canary Islands and Madeira vs. Selvagens.

**
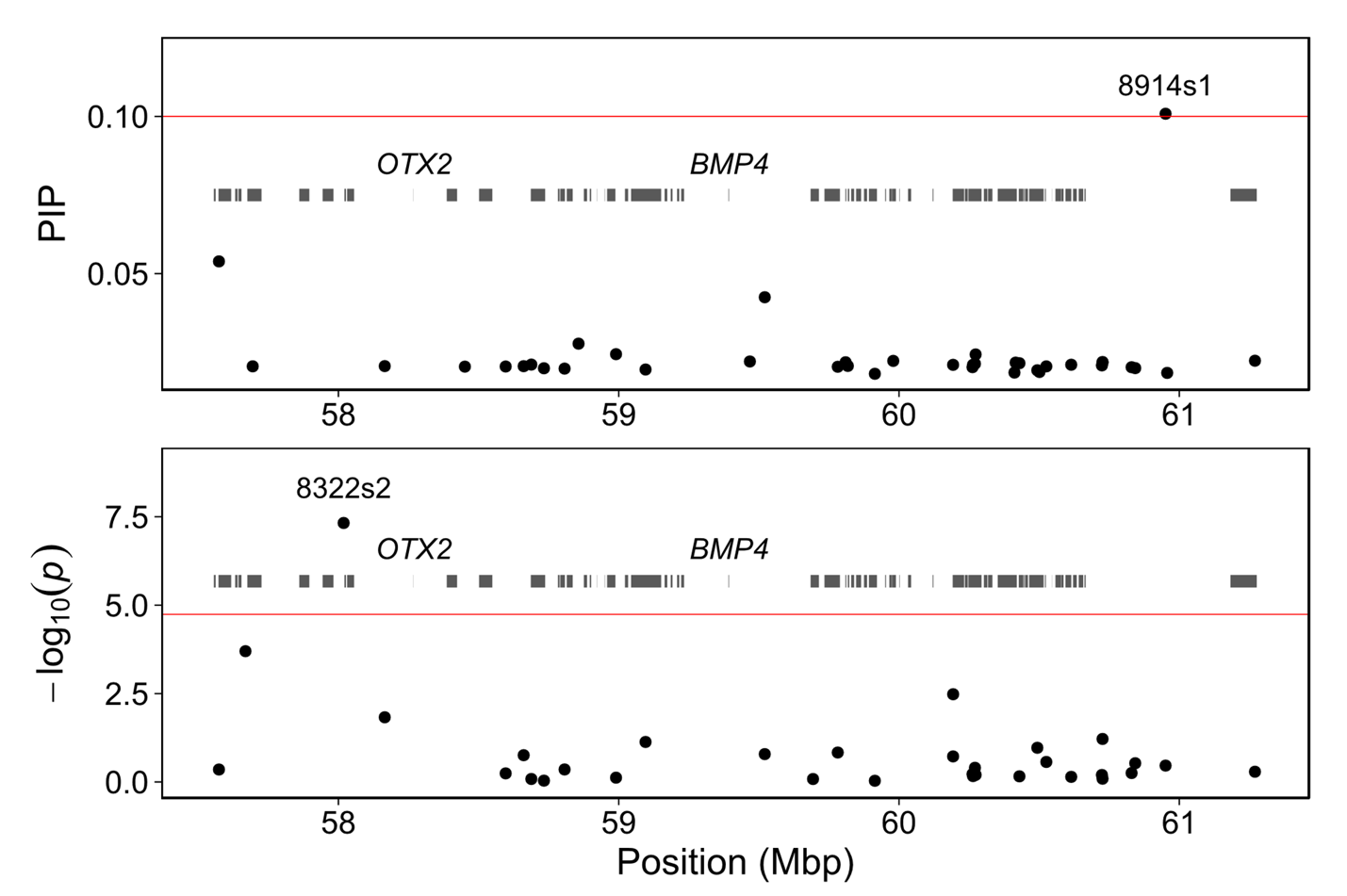
Figure S9** Region of interest for bill length on chromosome 5. Top panel: chromosome locations and posterior inclusion probabilities (PIP) for SNPs in the BSLMM bill length analysis in the "Canary Islands" dataset. Red line indicates PIP threshold of 0.1. Bottom panel: SNP chromosome locations and EigenGWAS Eigenvector 1 *p* values. Red line indicates Bonferroni-corrected *p* value threshold. Grey rectangles show location of genes within this region, with *OTX2* and *BMP4* labelled.

**
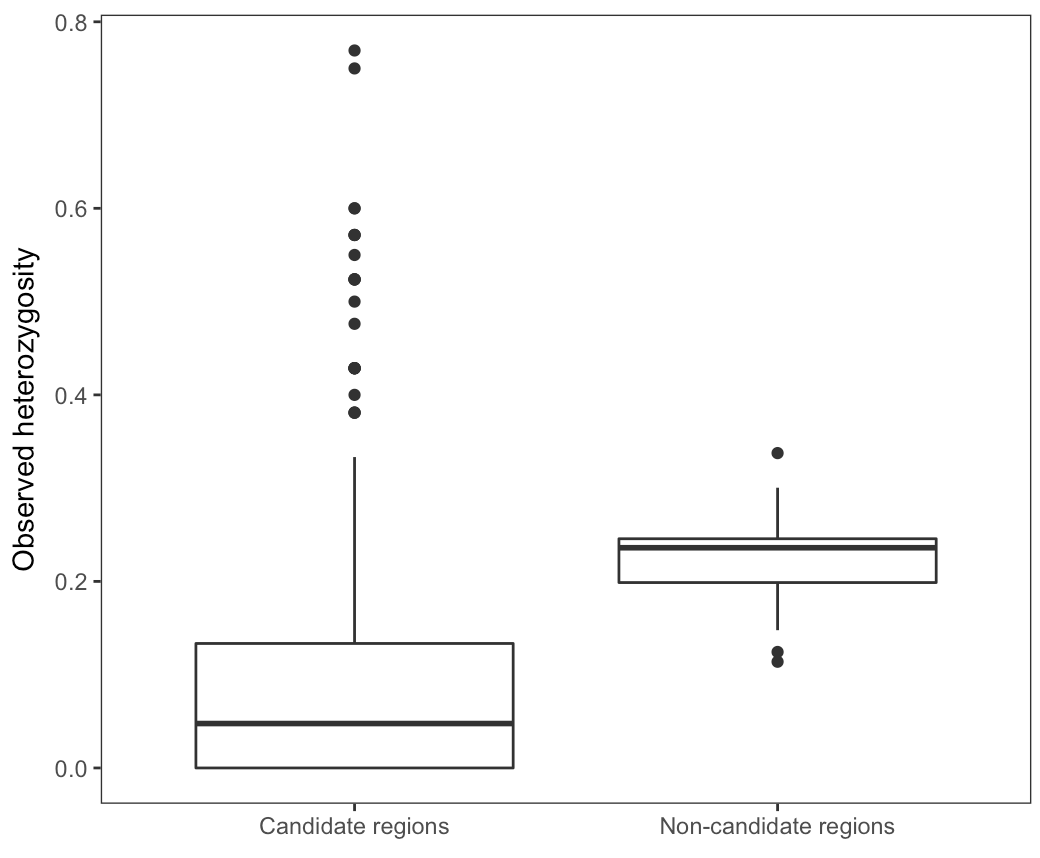
**

**Figure S10** Individual observed heterozygosity averaged across SNPs taken from EigenGWAS outliers (candidate regions - see main text) versus the rest of the genome (non-candidate regions).

**
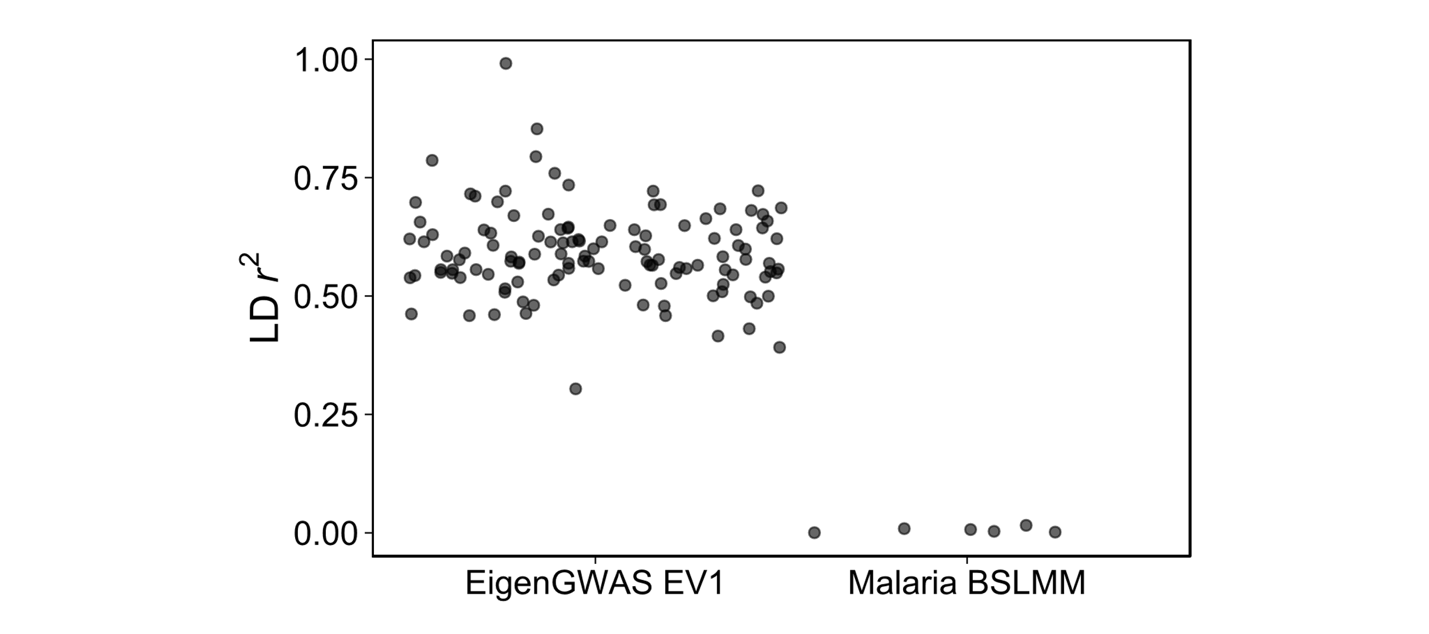
Figure S11** Linkage disequilibrium between all pairs of outlier SNPs from EigenGWAS Eigenvector 1 and malaria BSLMM analysis. Measures of LD for both sets of SNPs were calculated using the “Berthelot’s” dataset.
